# Nickel-Catalyzed Decarbonylative Stannylation of Acyl Fluorides under Ligand-Free Conditions

**DOI:** 10.3390/molecules24091671

**Published:** 2019-04-28

**Authors:** Xiu Wang, Zhenhua Wang, Li Liu, Yuya Asanuma, Yasushi Nishihara

**Affiliations:** 1 Graduate School of Natural Science and Technology, Okayama University, 3-1-1 Tsushimanaka, Kita-ku, Okayama 700-8530, Japan; p5ri81bx@s.okayama-u.ac.jp (X.W.); ptpl9ag1@s.okayama-u.ac.jp (Z.W.); p7b567xo@s.okayama-u.ac.jp (L.L.); p1y54tsv@s.okayama-u.ac.jp (Y.A.); 2 Research Institute for Interdisciplinary Science, Okayama University, 3-1-1 Tsushimanaka, Kita-ku, Okayama 700-8530, Japan

**Keywords:** nickel, acyl fluorides, stannylation, decarbonylation, carbon-tin bond formation

## Abstract

Nickel-catalyzed decarbonylative stannylation of acyl fluorides under ligand-free conditions was disclosed. A variety of aromatic acyl fluorides are capable of reacting with silylstannanes in the presence of cesium fluoride. A one-pot decarbonylative stannylation/Migita-Kosugi-Stille reaction of benzoyl fluoride, giving rise to the direct formation of the corresponding cross-coupled products, further demonstrated the synthetic utility of the present method. This newly developed methodology with a good functional-group compatibility via C–F bond cleavage and C–Sn bond formation under nickel catalysis opens a new area for the functionalization of acyl fluorides in terms of carbon-heteroatom bond formation.

## 1. Introduction

Acyl fluorides as one of carboxylic acid derivatives have attracted much attention in organic synthesis, due to their great stability, easy availability, and unique intrinsic nature [1,2,3]. Conventionally, transition metal-catalyzed transformations of acyl fluorides with organometallic reagents (Zn, Si, and B) have focused on the synthesis of biaryl ketones in a carbonyl-group retentive manner [4,5,6]. In a sharp contrast, recently, decarbonylative transformations of carboxylic acid derivatives [7,8], especially acyl fluorides have been studied intensively [9]. Sakai and Ogiwara have disclosed that the auxiliary ligand of the palladium catalyst can control the reaction type of reduction of acyl fluorides [10]. However, transition-metal catalyzed decarbonylative transformations of acyl fluorides are mainly C–C bond formation, such as palladium-catalyzed trifluoromethylation [11], nickel-catalyzed Suzuki-Miyaura reaction [12], iridium-catalyzed arylation via C–H bond activation [13], and our recent work on nickel-catalyzed ethylation with BEt_3_ [14] and DPPM-assisted methylenation with AlMe_3_ [15]. Therefore, the development of carbon-heteroatom bond-forming reactions of acyl fluorides are of great importance. Very recently, we successfully demonstrated the first nickel-catalyzed decarbonylative borylation of acyl fluorides with diboron, forming the C–B bond [16], and the related decarbonylative borylation catalyzed by palladium have been reported [17,18].

**Scheme 1:**
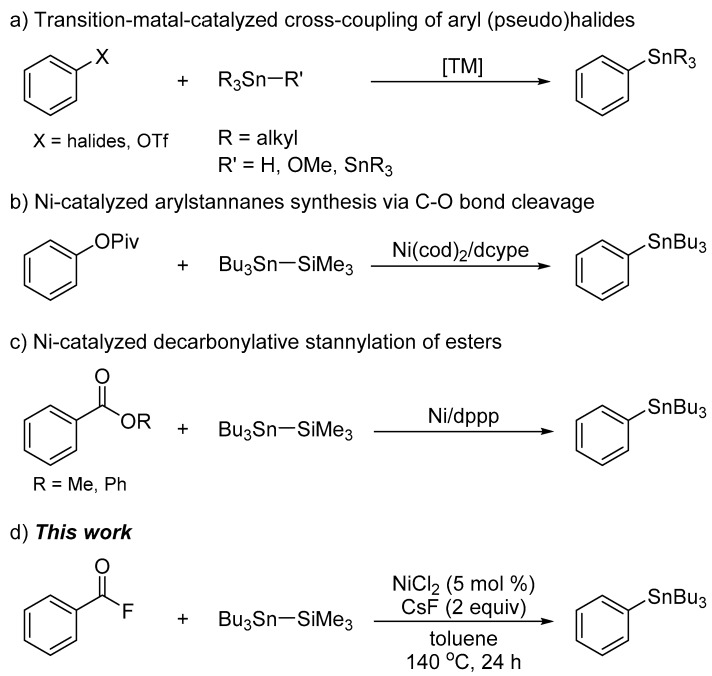
Various synthetic routes for arylstannanes.

Arylstannanes as one of common organometallic reagents are extensively applied in Migita-Kosugi-Stille reaction [19,20,21], which has been utilized as a powerful method for C–C bond formation, especially, in natural product synthesis [22,23,24]. Conventional synthetic methods of arylstannanes are the reactions of organometallic reagents such as arylzinc compounds with triorganotin halides [25,26,27]. Alternatively, catalytic cross-coupling reactions of aryl (pseudo)halides with tributyltin hydride [28], tributylstannyl methoxide [29], and hexaalkyldistannane [30,31] have been documented (Scheme 1a). Recent studies on arylstannanes synthesis utilizing air- and moisture-insensitive silylstannyl reagent, Bu_3_Sn-SiMe_3_, could prove that the C–O bond is also a powerful alternative to aryl halides (Scheme 1b) [32]. In addition, Rueping and co-workers developed nickel-catalyzed stannylation of aromatic esters in a decarbonylative manner (Scheme 1c) [33]. Although these methods have made a great contribution to the synthesis of arylstannanes, novel and practical methods to afford arylstannanes from more simple starting materials remain highly desirable. Herein, we report the first decarbonylative stannylation of acyl fluorides with Bu_3_Sn-SiMe_3_ catalyzed by air-stable and inexpensive nickel(II) chloride under ligand and additive-free conditions (Scheme 1d). 

## 2. Results and Discussion

We commenced our research by choosing benzoyl fluoride (**1a**) and 1.5 equiv of Bu_3_Sn-SiMe_3_ (**2**) as the model substrates, and the results are summarized in Table 1. Various transition metal sources were investigated to facilitate the decarbonylative stannylation reaction (entries 1–6). Among them, nickel(II) chloride displayed a superior result, affording the target product **3a** in 90% yield (entry 3). When cesium carbonate was employed in place of cesium fluoride, the yield of **3a** was dramatically dropped to 48% (entry 7) and no stannylation reaction occurred when potassium fluoride was used, along with silylstannane **2** recovered (entry 8). Additionally, amounts of **2** could be reduced to 1.2 equiv, which afforded 94% GC yield of **3a** (entries 3, 9, and 10). The yields of **3a** were slightly decreased as the reaction time was shortened (entries 9 vs. 11–13). When benzoyl chloride was employed instead of **1a**, **3a** was obtained in 56% yield, suggesting the unique feature of the present reaction of acyl fluorides.

A generality of the decarbonylative stannylation was examined with the optimized reaction conditions (Table 2). Acyl fluorides bearing electron-donating groups such as alkyl (**1b**–**1d**), phenyl (**1e**–**1g**), and alkoxy (**1h, 1i**) groups gave the corresponding products **3b**–**3i** in 56%–85% yields regardless of the substitution positions. Other oxygen-containing functional groups such as benzyloxy (**1j**) and acetal (**1k**) were also well tolerated during the reaction. Acyl fluorides bearing electron-withdrawing groups such as trifluoromethyl (**1l**) and fluoro (**1m, 1n**) groups were also well compatible. In particular, an aryl chloride skeleton (**1o**) is known to a reactive electrophile in some nickel-catalyzed cross-coupling reactions [29]. Although 4-bromo- and 4-iodobenzoyl fluorides were employed as the substrates, no trace of the desired products was detected, presumably due to the bromo and iodo groups are highly reactive under the present reaction conditions. Acyl fluorides with fused aromatic systems (**1p**–**1r**) afforded arylstannanes in moderate to good yields. Heterocycles including benzothiophene and quinoline yielded **3s** and **3t** in 62% and 84% yields, respectively. Unfortunately, however, the reactions employing surrogate aliphatic acyl fluorides gave no formation of the desired products.

**Scheme 2:**
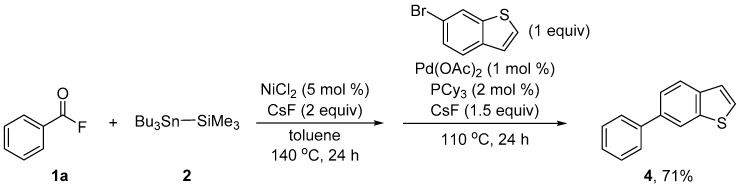
One-pot reaction of decarbonylative stannylation/Migita-Kosugi-Stille reaction of **1a**.

To demonstrate the synthetic utility of the present method, one-pot reaction of a successive decarbonylative stannylation/Migita-Kosugi-Stille reaction of **1a** was investigated (Scheme 2) [31]. To our delight, with the aid of the additional palladium catalyst into the reaction mixture, 71% yield of compound **4** was obtained. 

To gain more detailed insights into the reaction mechanism, some control experiments were carried out (Table 3). Although arylstannane **3a** was obtained in 94% yield, along with the formation of hexabutylditin (**5**; 13%) under the optimized reaction conditions (entry 1), indicating that nickel(II) chloride was reduced to Ni(0) species. This hypothesis was further proved by the reaction of **1a** with **2** in the absence of cesium fluoride (entry 2). Without nickel(II) chloride, no target product **3a** was formed, and **5** was obtained quantitatively (entry 3). In some stannylation reactions, hexabutyldistannane (**5**) could also be used as a stannylating reagent [30,31]. Thus, the reaction of **1a** (0.2 mmol) with **5** (0.24 mmol) was evaluated under identical reaction conditions. However, neither the desired product **3a** nor a viable acyl stannane was delivered, along with decomposition of **1a** and the remained **5** unreacted, which suggests that the once formed **5** never be involved into the catalytic cycle because of its lower reactivity (entry 4). 

**Scheme 3:**
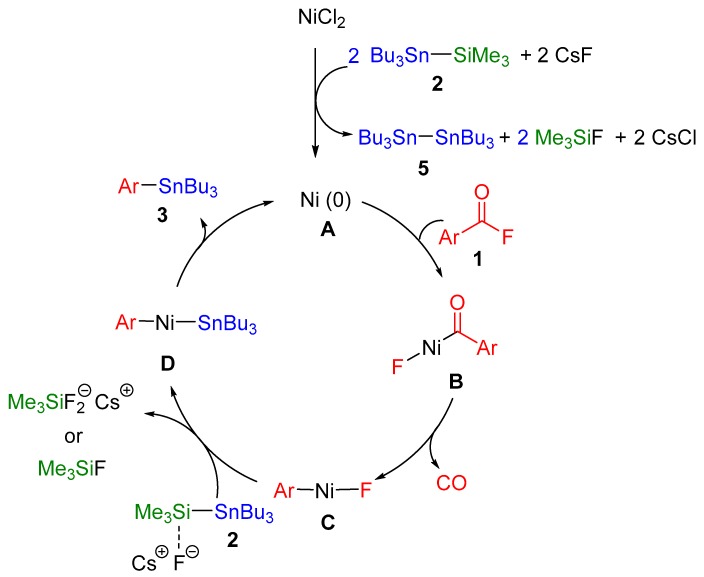
Proposed mechanism.

Our proposed mechanism of the present decarbonylative stannylation is outlined in Scheme 3. Combining the related references [12,34] with our previous work [16], it is assumed that oxidative addition of acyl fluorides **1** to Ni(0) species **A**, derived from reduction of Ni(II) chloride with **2**, yields acyl nickel(II) species **B**. Subsequently, decarbonylation of **B** delivers arylnickel(II) species **C** [12,16]. Transmetalation between the complex **C** and activated silylstannane **2** by cesium fluoride affords complex **D** [32]. Following reductive elimination gives the target product arylstannanes **3**, regenerating Ni(0) species **A**. 

## 3. Experimental Sections

### 3.1. General

Unless otherwise noted, all the reactions were carried out under an argon atmosphere using standard Schlenk techniques. Glassware was dried in an oven (150 °C) and heated under reduced pressure prior to use. Solvents were employed as eluents for all other routine operation, as well as dehydrated solvent were purchased from commercial suppliers and employed without any further purification. For thin layer chromatography (TLC) analyses throughout this work, Merck precoated TLC plates (silica gel 60 GF254, 0.25 mm) were used. Silica gel column chromatography was carried out using silica gel 60 N (spherical, neutral, 40–100 μm) from Kanto Chemicals Co., Inc. (Tokyo, Japan). NMR spectra (^1^H, ^13^C{^1^H} and ^19^F{^1^H}) were recorded on Varian INOVA-600 (600 MHz), Mercury-400 (400 MHz), or 300-NMR ASW (300 MHz) spectrometers (Agilent Technologies International Japan, Ltd., Tokyo, Japan). Chemical shifts (*δ*) are in parts per million relative to CDCl_3_ at 7.26 ppm for ^1^H and at 77.16 ppm for ^13^C{^1^H}. The ^19^F{^1^H} NMR spectra were measured by using CCl_3_F (*δ* = 0.00 ppm) as an external standard. The NMR yields were determined using dibromomethane as an internal standard. The GC yields were determined by GC analysis of the crude mixture, using *n*-dodecane as an internal standard. GC analyses were performed on a Shimadzu GC-14A equipped with a flame ionization detector using Shimadzu Capillary Column (CBP1-M25-025) and Shimadzu C-R6A-Chromatopac integrator (Kyoto, Japan). Infrared spectra were recorded on a SHIMADZU IRPrestige-21 spectrophotometer. Elemental analyses were carried out with a Perkin-Elmer 2400 CHN elemental analyzer (Perkin-Elmer, Waltham, MA, USA) at Okayama University. ^1^H NMR, ^13^C{^1^H} NMR, ^19^F{^1^H} NMR spectra of the compounds **1t**, **2**, **3a**–**t** and **4** can be found at the Supplementary Materials.

### 3.2. Experimental Method

#### 3.2.1. Representative Procedure for the Synthesis of Acyl Fluorides from Acyl Chlorides

To a 50 mL of Schlenk tube charged with a magnetic stir bar, were successively added acyl chloride (4.0 mmol), 18-crown-6 (52.9 mg, 0.2 mmol, 5 mol %), KF (2.32 g, 40 mmol, 10 equiv), and THF (20 mL). After the reaction was stirred at 40 °C for 24 h, insoluble inorganic solid (KF or KCl) was filtered, and the volatiles were concentrated using a rotary evaporator. The crude product was purified by bulb-to-bulb distillation to afford the corresponding acyl fluorides **1** [35].

#### 3.2.2. Representative Procedure for the Synthesis of Acyl Fluorides from Carboxylic Acids

To a 20 mL of Schlenk tube charged with a magnetic stir bar, were successively added carboxylic acid (3.0 mmol) and CH_2_Cl_2_ (15 mL). After the mixture was stirred at 0 °C for 30 min, Deoxo-Fluor^®^ reagent (608 μL, 3.3 mmol, 1.1 equiv) was slowly added to the reaction mixture. After the reaction mixture was stirred at 0 °C for 30 min, the solution was slowly poured into saturated NaHCO_3_, extracted with CH_2_Cl_2_ (3 × 15 mL), and dried over MgSO_4_. The crude product was purified by flash chromatography on silica gel to afford the corresponding acyl fluorides **1** [36].

#### 3.2.3. Synthesis of Trimethyl(tributylstannyl)silane (**2**)

To a solution of naphthalene (51.3 mg, 0.4 mmol) in THF (16 mL), was added lithium clippings (84 mg, 12 mmol) under an argon atmosphere. During the resulting mixture was stirred at room temperature for 1 h, the color turned to dark green. Then, hexabutylditin (2.02 mL, 4 mmol) was added dropwise and the mixture was stirred at room temperature for 3 h. The resulting solution (16 mL) was transferred via cannula to a Schlenk tube under Ar and then stored at room temperature. A THF solution prepared as described above was added via a cannula into the stirred solution of chlorotrimethylsilane (951 mg, 8.8 mmol) in THF at 0 °C. The reaction was stirred at room temperature overnight followed by extraction with hexane. The organic phase was washed with brine and dried over Na_2_SO_4_. Removal of the solvent and purification by bulb-to-bulb distillation under reduced pressure provided Bu_3_Sn-SiMe_3_ as a colorless oil [37].

#### 3.2.4. Representative Procedure for Ni-catalyzed Decarbonylative Stannylation of Acyl Fluorides

A 20 mL dried Schlenk tube containing a stirring bar and CsF (60.8 mg, 0.4 mmol, 2 equiv) was dried with a heat gun under reduced pressure and filled with Ar after cooling to room temperature. To this vessel, were added NiCl_2_ (1.3 mg, 0.01 mmol, 5 mol %), toluene (1 mL), acyl fluorides (**1**) (0.2 mmol, 1 equiv) and trimethyl(tributylstannyl)silane (**2**) (87.2 mg, 0.24 mmol, 1.2 equiv). The mixture was heated at 140 °C with stirring for 24 h. The solution was then cooled to room temperature, and the solvent was removed under vacuum. The decarbonylative stannylation products **3** were purified by flash column chromatography on silica gel. 

#### 3.2.5. One-Pot Decarbonylative Stannylation/Migita-Kosugi-Stille Cross-Coupling Reaction of **1a**

A 20 mL dried Schlenk tube containing a stirring bar and CsF (60.8 mg, 0.4 mmol, 2 equiv) was dried with a heat gun under reduced pressure and filled with Ar after cooling to room temperature. To this vessel, were added with NiCl_2_ (1.3 mg, 0.01 mmol, 5 mol %), toluene (1 mL), benzoyl fluoride (**1a**) (24.8 mg, 0.2 mmol), and trimethyl(tributylstannyl)silane (**2**) (87.2 mg, 0.24 mmol, 1.2 equiv). The mixture was heated at 140 °C with stirring for 24 h. The solution was then cooled to room temperature. 6-Bromobenzo[*b*]thiophene (42.6 mg, 0.2 mmol, 1 equiv), palladium acetate (0.4 mg, 0.002 mmol, 1 mol %), tricyclohexylphosphine (1.1 mg, 0.004 mmol, 2 mol %), and anhydrous cesium fluoride (45.6 mg, 0.3 mmol, 1.5 equiv) were added to the reaction mixture. The mixture was heated at 110 °C with stirring. After 24 h, the reaction mixture was cooled, the volatiles were evaporated under reduced pressure. The product was purified by flash chromatography on silica gel by elution with hexane, compound **4** was obtained in 71% yield (30 mg, 0.14 mmol) as white solid [31].

### 3.3. Characterization Data of Starting Materials and Products

*Quinoline-6-carbonyl fluoride* (**1t**). Yield: 55% (288.8 mg); white solid; melting point: 104–105 °C; ^1^H NMR (400 MHz, CDCl_3_) *δ* 7.53–7.56 (m, 1H), 8.20–8.25 (m, 2H), 8.30 (dd, *J* = 8.4, 1.9 Hz, 1H), 8.62 (d, *J* = 1.9 Hz, 1H), 9.08 (dd, *J* = 4.3, 1.9 Hz, 1H); ^13^C{^1^H} NMR (101 MHz, CDCl_3_) δ 122.5, 123.1, 127.4, 129.4 (d, *J*_C-F_ = 4.0 Hz), 130.8 (d, *J*_C-F_ = 1.4 Hz), 133.9 (d, *J*_C-F_ = 3.0 Hz), 137.6, 150.7, 153.8, 156.9 (d, *J_C_*_-F_ = 344.2 Hz); ^19^F{^1^H} NMR (282 MHz, CDCl_3_) δ 18.9; FT-IR (neat, cm^−1^): 735 (s), 781 (s), 854 (s), 1011 (s), 1045 (s), 1171 (s), 1231 (s), 1622 (s), 1805 (s); Anal. Calcd for C_14_H_9_FO_3_: C, 68.57; H, 3.45; N, 8.00%. Found: C, 68.50; H, 3.23; N, 7.95%.

*Trimethyl(tributylstannyl)silane* (**2**) [37]. Yield: 92% (2.67 g); colorless oil; ^1^H NMR (600 MHz, CDCl_3_) δ 0.23 (s, *J*_H-Sn_ = 26.4 Hz, 9H), 0.84–0.90 (m, 15H), 1.29 (sext, *J* = 7.3 Hz, 6H), 1.44–1.48 (m, 6H).

*Tributyl(phenyl)stannane* (**3a**) [33]. Yield: 90% (66.1 mg); colorless oil; ^1^H NMR (600 MHz, CDCl_3_) δ 0.91–0.95 (m, 9H), 1.03–1.16 (m, *J*_H-Sn_ = 54.6 Hz, 6H), 1.33–1.41 (m, 6H), 1.53–1.63 (m, 6H), 7.32–7.36 (m, 3H), 7.46–7.54 (m, *J*_H-Sn_ = 36.4 Hz, 2H).

*Tributyl(p-tolyl)stannane* (**3b**) [33]. Yield: 81% (61.8 mg); colorless oil; ^1^H NMR (600 MHz, CDCl_3_) δ 0.90 (t, *J* = 7.2 Hz, 9H), 1.00–1.10 (m, *J*_H-Sn_ = 52.4 Hz, 6H), 1.31–1.38 (m, 6H), 1.48–1.59 (m, 6H), 2.35 (s, 3H), 7.17 (d, *J* = 7.2 Hz, 2H), 7.37 (d, *J* = 7.8 Hz, *J*_H-Sn_ = 35.8 Hz, 2H).

*Tributyl(o-tolyl)stannane* (**3c**) [31]. Yield: 63% (48.0 mg); colorless oil; ^1^H NMR (400 MHz, CDCl_3_) δ 0.90 (t, *J* = 7.2 Hz, 9H), 1.01–1.14 (m, *J*_H-Sn_ = 50.4 Hz, 6H), 1.34 (sext, *J* = 7.2 Hz, 6H), 1.47–1.58 (m, 6H), 2.40 (s, 3H), 7.12–7.17 (m, 1H), 7.17–7.25 (m, 2H), 7.40 (d, *J* = 6.8 Hz, *J*_H-Sn_ = 42.8 Hz, 1H).

*Tributyl(4-butylphenyl)stannane* (**3d**). Yield: 67% (56.7 mg); colorless oil; ^1^H NMR (400 MHz, CDCl_3_) δ 0.89 (t, *J* = 7.6 Hz, 9H), 0.94 (t, *J* = 7.6 Hz, 3H), 0.99–1.12 (m, *J*_H-Sn_ = 50.8 Hz, 6H), 1.29–1.40 (m, 8H), 1.50–1.64 (m, 8H), 2.60 (t, *J* = 7.8 Hz, 2H), 7.14–7.19 (m, 2H), 7.37 (d, *J* = 6.4 Hz, *J*_H-Sn_ = 39.2 Hz, 2H); ^13^C{^1^H} NMR (101 MHz, CDCl_3_) δ 9.7 (*J*_C-Sn_ = 338.7 Hz), 13.8, 14.1, 22.6, 27.6 (*J*_C-Sn_ = 57.2 Hz), 29.3 (*J*_C-Sn_ = 19.8 Hz), 33.8, 35.8, 128.3 (*J*_C-Sn_ = 41.2 Hz), 136.5 (*J*_C-Sn_ = 31.3 Hz), 138.3, 142.7; FT-IR (neat, cm^−1^): 729 (m), 748 (m), 1070 (m), 1045 (m), 1377 (m), 1458 (m), 2855 (m), 2872 (m), 2928 (m), 2959 (m); Anal. Calcd for C_22_H_40_Sn: C, 62.43; H, 9.53%. Found: C, 62.30; H, 9.55%.

*[1,1′-Biphenyl]-4-yltributylstannane* (**3e**) [33]. Yield: 82% (72.7 mg); colorless oil; ^1^H NMR (600 MHz, CDCl_3_) δ 0.93 (t, *J* = 7.5 Hz, 9H), 1.03–1.18 (m, *J*_H-Sn_ = 43.2 Hz, 6H), 1.38 (sext, *J* = 7.5 Hz, 6H), 1.55–1.66 (m, 6H), 7.35–7.39 (m, 1H), 7.45–7.48 (m, 2H), 7.53–7.60 (m, 4H), 7.62–7.64 (m, 2H).

*[1,1′-Biphenyl]-3-yltributylstannane* (**3f**) [33]. Yield: 56% (49.6 mg); colorless oil; ^1^H NMR (600 MHz, CDCl_3_) δ 0.91 (t, *J* = 7.5 Hz, 9H), 1.06–1.16 (m, *J*_H-Sn_ = 50.8 Hz, 6H), 1.33–1.38 (m, 6H), 1.54–1.63 (m, 6H), 7.35–7.38 (m, 1H), 7.40–7.48 (m, 4H), 7.51–7.54 (m, 1H), 7.58–7.62 (m, 2H), 7.63–7.71 (d, *J* = 1.8 Hz, *J*_H-Sn_ = 40.2 Hz, 1H).

*[1,1′-Biphenyl]-2-yltributylstannane* (**3g**) [33]. Yield: 85% (75.4 mg); colorless oil; ^1^H NMR (600 MHz, CDCl_3_) δ 0.69–0.77 (m, *J*_H-Sn_ = 51.0 Hz, 6H), 0.84 (t, *J* = 7.3 Hz, 9H), 1.23 (sext, *J* = 7.8 Hz, 6H), 1.32–1.38 (m, 6H), 7.32–7.35 (m, 3H), 7.36–7.39 (m, 3H), 7.39–7.42 (m, 2H), 7.56 (d, *J* = 7.3 Hz, *J*_H-Sn_ = 40.8 Hz, 1H).

*Tributyl(4-methoxyphenyl)stannane* (**3h**) [31]. Yield: 64% (50.8 mg); colorless oil; ^1^H NMR (400 MHz, CDCl_3_) δ 0.89 (t, *J* = 7.2 Hz, 9H), 0.96–1.09 (m, *J*_H-Sn_ = 51.2 Hz, 6H), 1.33 (sext, *J* = 7.6 Hz, 6H), 1.48–1.59 (m, 6H), 3.81 (s, 3H), 6.91 (d, *J* = 8.0 Hz, 2H), 7.38 (d, *J* = 8.0 Hz, *J*_H-Sn_ = 37.2 Hz, 2H).

*(4-Butoxyphenyl)tributylstannane* (**3i**). Yield: 61% (53.6 mg); colorless oil; ^1^H NMR (600 MHz, CDCl_3_) δ 0.89 (t, *J* = 7.2 Hz, 9H), 0.98 (t, *J* = 7.2 Hz, 3H), 1.01–1.04 (m, *J*_H-Sn_ = 51.6 Hz, 6H), 1.33 (sext, *J* = 7.8 Hz, 6H), 1.47–1.58 (m, 8H), 1.75–1.80 (m, 2H), 3.96 (t, *J* = 6.6 Hz, 2H), 6.90 (d, *J* = 8.5 Hz, *J*_H-Sn_ = 61.8 Hz, 2H), 7.36 (d, *J* = 8.5 Hz, *J*_H-Sn_ = 42.6 Hz, 2H); ^13^C{^1^H} NMR (151 MHz, CDCl_3_) δ 9.7 (*J_C-Sn_* = 333.0 Hz), 13.9, 14.0, 19.4, 27.6 (*J*_C-Sn_ = 55.5 Hz), 29.2 (*J*_C-Sn_ = 20.8 Hz), 31.5, 67.4, 114.6 (*J*_C-Sn_ = 43.9 Hz), 131.8, 137.6 (*J*_C-Sn_ = 34.7 Hz), 159.4; FT-IR (neat, cm^−1^): 671 (m), 754 (m), 1072 (m), 1130 (m), 1207 (m), 1242 (m), 1273 (m), 1464 (m), 1495 (m), 1585 (m), 2855 (m), 2872 (m), 2928 (m), 2959 (m); Anal. Calcd for C_22_H_40_OSn: C, 60.15; H, 9.18%. Found: C, 60.09; H, 9.32%.

*(4-(Benzyloxy)phenyl)tributylstannane* (**3j**) [38]. Yield: 50% (47.3 mg); colorless oil; ^1^H NMR (600 MHz, CDCl_3_) δ 0.90 (t, *J* = 7.2 Hz, 9H), 0.98–1.10 (m, *J*_H-Sn_ = 51.0 Hz, 6H), 1.34 (sext, *J* = 7.2 Hz, 6H), 1.52–1.57 (m, 6H), 5.07 (s, 2H), 7.00 (d, *J* = 8.5 Hz, 2H), 7.32–7.36 (m, 1H), 7.37–7.43 (m, 4H), 7.46 (d, *J* = 7.2 Hz, 2H).

*Benzo[d][1,3]dioxol-5-yltributylstannane* (**3k**) [29]. Yield: 87% (71.5 mg); colorless oil; ^1^H NMR (600 MHz, CDCl_3_) δ 0.90 (t, *J* = 7.2 Hz, 9H), 0.99–1.08 (m, *J*_H-Sn_ = 49.8 Hz, 6H), 1.33 (sext, *J* = 7.8 Hz, 6H), 1.50–1.58 (m, 6H), 5.92 (s, 2H), 6.86 (d, *J* = 7.4 Hz, 1H), 6.90 (d, *J* = 7.5 Hz, 1H), 6.94 (s, *J*_H-Sn_ = 37.2 Hz, 1H).

*Tributyl(4-(trifluoromethyl)phenyl)stannane* (**3l**) [33]. Yield: 61% (53.1 mg); colorless oil; ^1^H NMR (600 MHz, CDCl_3_) δ 0.89 (m, *J* = 7.2 Hz, 9H), 1.04–1.15 (m, *J*_H-Sn_ = 50.6 Hz, 6H), 1.33 (sext, *J* = 7.2 Hz, 6H), 1.50–1.57 (m, 6H), 7.55 (d, *J* = 8.0 Hz, 2H), 7.58 (d, *J* = 8.0 Hz, *J*_H-Sn_ = 34.8 Hz, 2H).

*Tributyl(4-fluorophenyl)stannane* (**3m**) [33]. Yield: 86% (66.2 mg); colorless oil; ^1^H NMR (400 MHz, CDCl_3_) δ 0.89 (t, *J* = 7.3 Hz, 9H), 0.98–1.12 (m, *J*_H-Sn_ = 51.0 Hz, 6H), 1.33 (sext, *J* = 8.0 Hz, 6H), 1.47–1.60 (m, 6H), 7.00–7.08 (m, 2H), 7.35–7.48 (m, 2H).

*Tributyl(4′-fluoro-[1,1′-biphenyl]-4-yl)stannane* (**3n**) [33]. Yield: 72% (66.4 mg); colorless oil; ^1^H NMR (600 MHz, CDCl_3_) δ 0.91 (t, *J* = 7.2 Hz, 9H), 1.05–1.15 (m, *J*_H-Sn_ = 50.1 Hz, 6H), 1.36 (sext, *J* = 7.2 Hz, 6H), 1.54–1.62 (m, 6H), 7.13 (dd, *J* = 8.7 Hz, *J_F-H_* = 8.7 Hz, 2H), 7.51–7.53 (m, 2H), 7.54–7.57 (m, 4H).

*Tributyl(4-chlorophenyl)stannane* (**3o**) [37]. Yield: 90% (72.3 mg); colorless oil; ^1^H NMR (600 MHz, CDCl_3_) δ 0.88 (t, *J* = 7.5 Hz, 9H), 1.00–1.10 (m, *J*_H-Sn_ = 50.1 Hz, 6H), 1.32 (sext, *J* = 7.2 Hz, 6H), 1.47–1.56 (m, 6H), 7.28–7.33 (m, 2H), 7.34–7.42 (m, *J*_H-Sn_ = 36.0 Hz, 2H).

*Tributyl(naphthalen-1-yl)stannane* (**3p**) [33]. Yield: 51% (42.6 mg); colorless oil; ^1^H NMR (600 MHz, CDCl_3_) δ 0.88 (t, *J* = 7.2 Hz, 9H), 1.19–1.22 (m, *J*_H-Sn_ = 50.4 Hz, 6H), 1.35 (sext, *J* = 7.8 Hz, 6H), 1.54–1.60 (m, 6H), 7.42–7.51 (m, 3H), 7.63 (d, *J* = 6.6 Hz, *J*_H-Sn_ = 46.2 Hz, 1H), 7.77 (d, *J* = 7.8 Hz, 1H), 7.81 (d, *J* = 7.8 Hz, 1H), 7.85 (d, *J* = 7.2 Hz, 1H).

*Tributyl(naphthalen-2-yl)stannane* (**3q**) [33]. Yield: 79% (65.9 mg); colorless oil; ^1^H NMR (600 MHz, CDCl_3_) δ 0.92 (m, *J* = 7.2 Hz, 9H), 1.09–1.21 (m, *J*_H-Sn_ = 50.1 Hz, 6H), 1.38 (sext, *J* = 7.2 Hz, 6H), 1.56–1.65 (m, 6H), 7.44–7.51 (m, 2H), 7.59 (d, *J* = 8.1 Hz, *J*_H-Sn_ = 33.0 Hz, 1H), 7.79–7.87 (m, 3H), 7.96 (s, *J*_H-Sn_ = 44.4 Hz, 1H).

*Tributyl(9H-fluoren-1-yl)stannane* (**3r**). Yield: 82% (74.7 mg); colorless oil; ^1^H NMR (600 MHz, CDCl_3_) δ 0.90 (t, *J* = 7.2 Hz, 9H), 1.11–1.23 (m, *J*_H-Sn_ = 51.0 Hz, 6H), 1.36 (sext, *J* = 7.2 Hz, 6H), 1.52–1.62 (m, 6H), 3.85 (s, 2H), 7.30–7.42 (m, 4H), 7.57 (d, *J* = 7.5 Hz, 1H), 7.76 (dd, *J* = 7.2, 1.2 Hz, 1H), 7.80 (d, *J* = 7.8 Hz, 1H); ^13^C{^1^H} NMR (151 MHz, CDCl_3_) δ 9.8 (*J*_C-Sn_ = 338.7 Hz), 13.8, 27.6 (*J*_C-Sn_ = 60.1 Hz), 29.4 (*J*_C-Sn_ = 19.6 Hz), 39.4, 119.9, 120.0, 125.0, 126.3, 126.6, 126.9, 135.0, 138.3, 140.3, 142.2, 143.0, 150.8; FT-IR (neat, cm^−1^): 731 (m), 752 (m), 1207 (s), 1220 (m), 1225 (m), 1456 (m), 1464 (m), 1695 (m), 2855 (m), 2865 (m), 2926 (m), 2959 (m); Anal. Calcd for C_25_H_36_Sn: C, 65.95; H, 7.97%. Found: C, 66.06; H, 8.20%.

*Benzo[b]thiophen-2-yltributylstannane* (**3s**) [39]. Yield: 62% (52.5 mg); colorless oil; ^1^H NMR (600 MHz, CDCl_3_) δ 0.91 (t, *J* = 7.2 Hz, 9H), 1.12–1.20 (m, *J*_H-Sn_ = 52.2 Hz, 6H), 1.36 (sext, *J* = 7.2 Hz, 6H), 1.56–1.64 (m, 6H), 7.27–7.29 (m, 1H), 7.32 (t, *J* = 6.6 Hz, 1H), 7.39 (s, *J*_H-Sn_ = 24.0 Hz, 1H), 7.82 (d, *J* = 8.4 Hz, 1H), 7.89 (d, *J* = 7.8 Hz, 1H).

*6-(Tributylstannyl)quinoline* (**3t**) [32]. Yield: 84% (70.3 mg); colorless oil; ^1^H NMR (600 MHz, CDCl_3_) δ 0.89 (t, *J* = 7.5 Hz, 9H), 1.08–1.20 (m, *J*_H-Sn_ = 51.0 Hz, 6H), 1.36 (sext, *J* = 7.8 Hz, 6H), 1.53–1.64 (m, 6H), 7.39 (dd, *J* = 8.4, 4.2 Hz, 1H), 7.81 (d, *J* = 7.8 Hz, *J*_H-Sn_ = 31.5 Hz, 1H), 7.91 (s, *J*_H-Sn_ = 42.0 Hz, 1H), 8.05 (d, *J* = 8.4 Hz, 1H), 8.13 (d, *J* = 8.4 Hz, 1H), 8.89 (dd, *J* = 8.4, 1.2 Hz, 1H).

*6-Phenylbenzo[b]thiophene* (**4**) [40]. Yield: 71% (30.0 mg); white solid; ^1^H NMR (400 MHz, CDCl_3_) δ 7.35–7.42 (m, 2H), 7.46–7.51 (m, 3H), 7.61 (dd, *J* = 8.4, 1.8 Hz, 1H), 7.66–7.90 (m, 2H), 7.95 (d, *J* = 8.4, 1H), 8.04 (d, *J* = 1.8 Hz, 1H).

## 4. Summary

In summary, we have developed an efficient and convenient method for the inexpensive NiCl_2_-catalyzed decarbonylative stannylation of a series of acyl fluorides, which is notable for being both ligand and additive-free. A one-pot decarbonylative stannylation/Migita-Kosugi-Stille reaction further demonstrated the synthetic applicability of our protocol because the isolation of toxic organotin compounds is not necessary. This study can expand the chemistry of acyl fluorides in terms of carbon-heteroatom bond formations.

## Figures and Tables

**Table 1:**
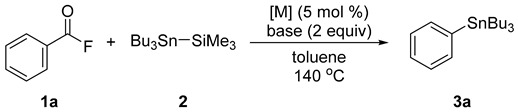
Optimization of the reaction conditions.

Entry	[M]	Base	2 (equiv)	Time (h)	Yield of 3a (%) ^1^
1	FeCl_2_	CsF	1.5	24	29
2	CoCl_2_	CsF	1.5	24	21
3	NiCl_2_	CsF	1.5	24	90
4	NiBr_2_	CsF	1.5	24	68
5	Ni(cod)_2_	CsF	1.5	24	16
6	PdCl_2_	CsF	1.5	24	4
7	NiCl_2_	Cs_2_CO_3_	1.5	24	48
8	NiCl_2_	KF	1.5	24	0
9	NiCl_2_	CsF	1.2	24	94 (90)
10	NiCl_2_	CsF	1.0	24	58
11	NiCl_2_	CsF	1.2	18	91
12	NiCl_2_	CsF	1.2	12	87
13	NiCl_2_	CsF	1.2	6	76

^1^ Determined by GC analysis of the crude mixture using *n*-dodecane as an internal standard. An isolated yield is given in parentheses.

**Table 2:**
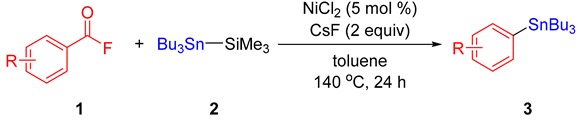
Decarbonylative stannylation of acyl fluorides ^a,b^.

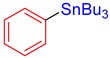 **3a**, 90%	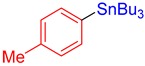 **3b**, 81%	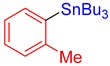 **3c**, 63%	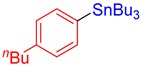 **3d**, 67%
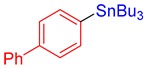 **3e**, 82%	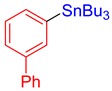 **3f**, 56%	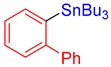 **3g**, 85%	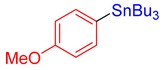 **3h**, 64%
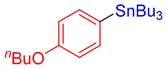 **3i**, 61%	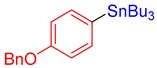 **3j**, 50%	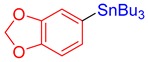 **3k**, 87%	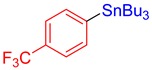 **3l**, 61%
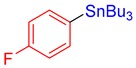 **3m**, 86%	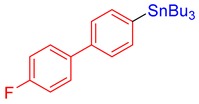 **3n**, 72%	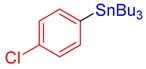 **3o**, 90%	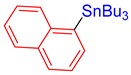 **3p**, 51%
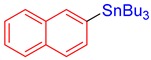 **3q**, 79%	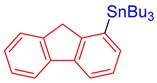 **3r**, 82%	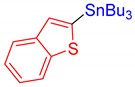 **3s**, 62%	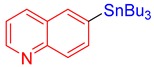 **3t**, 84%

^a^*Reaction conditions*: **1** (0.2 mmol), **2** (0.24 mmol), NiCl_2_ (0.01 mmol), CsF (0.4 mmol), toluene (1 mL), 140 °C, 24 h. ^b^ Isolated yields.

**Table 3:**
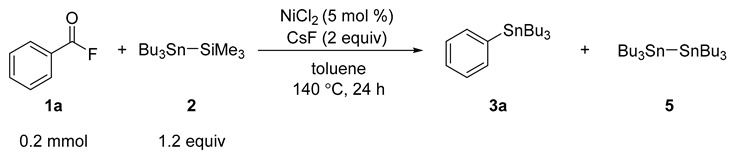
Control experiments for Ni-catalyzed decarbonylative stannylation.

Entry	Deviation from Standard Conditions	GC Yield (%) ^1^		
		2	3a	5
1	none	0	94	13
2	without CsF	120	0	0
3	without NiCl_2_	0	0	63
4 ^2^	**5** instead of **2**	-	0	>99

^1^ Determined by GC analysis of the crude mixture, using *n*-dodecane as an internal standard. ^2^ 4 (0.24 mmol) was added.

## Supplementary Materials

The following are available online. ^1^H NMR, ^13^C{^1^H} NMR, ^19^F{^1^H} NMR spectra of representative starting materials and final products.
