# Supplementary material for: Nickel-Catalyzed Decarbonylative Stannylation of Acyl Fluorides under Ligand-Free Conditions
Source: Molecules. 2019 Apr 28;24(9):1671. doi: 10.3390/molecules24091671 (PMC6539589; doi:10.3390/molecules24091671)
Supplement: Supplementary file 1 [file molecules-24-01671-s001.pdf]

Supplementary Materials

Nickel-Catalyzed Decarbonylative Stannylation of Acyl Fluorides under Ligand-Free Conditions

Xiu Wang[1], Zhenhua Wang[1], Li Liu[1], Yuya Asanuma[1], and Yasushi Nishihara[2,*]

[1] Graduate School of Natural Science and Technology, Okayama University, 3-1-1 Tsushimanaka, Kita-ku, Okayama 700-8530, Japan; p5ri81bx@s.okayama-u.ac.jp (X.W.); ptpl9ag1@s.okayama-u.ac.jp (Z.W.); p7b567xo@s.okayama-u.ac.jp (L.L.); p1y54tsv@s.okayama-u.ac.jp (Y.A.)

[2] Research Institute for Interdisciplinary Science, Okayama University, 3-1-1 Tsushimanaka, Kita-ku, Okayama 700-8530, Japan; ynishiha@okayama-u.ac.jp (Y.N.)

* Correspondence: ynishiha@okayama-u.ac.jp; Tel.: +81-86-251-7855

Copies of NMR Spectra

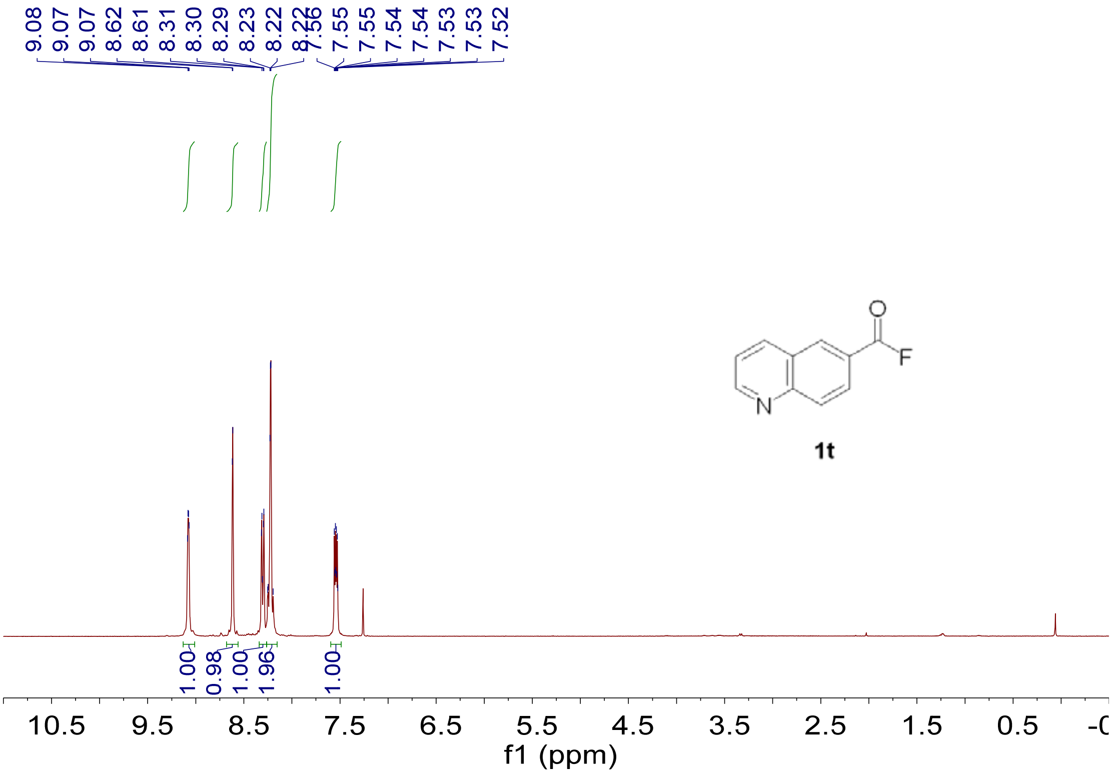

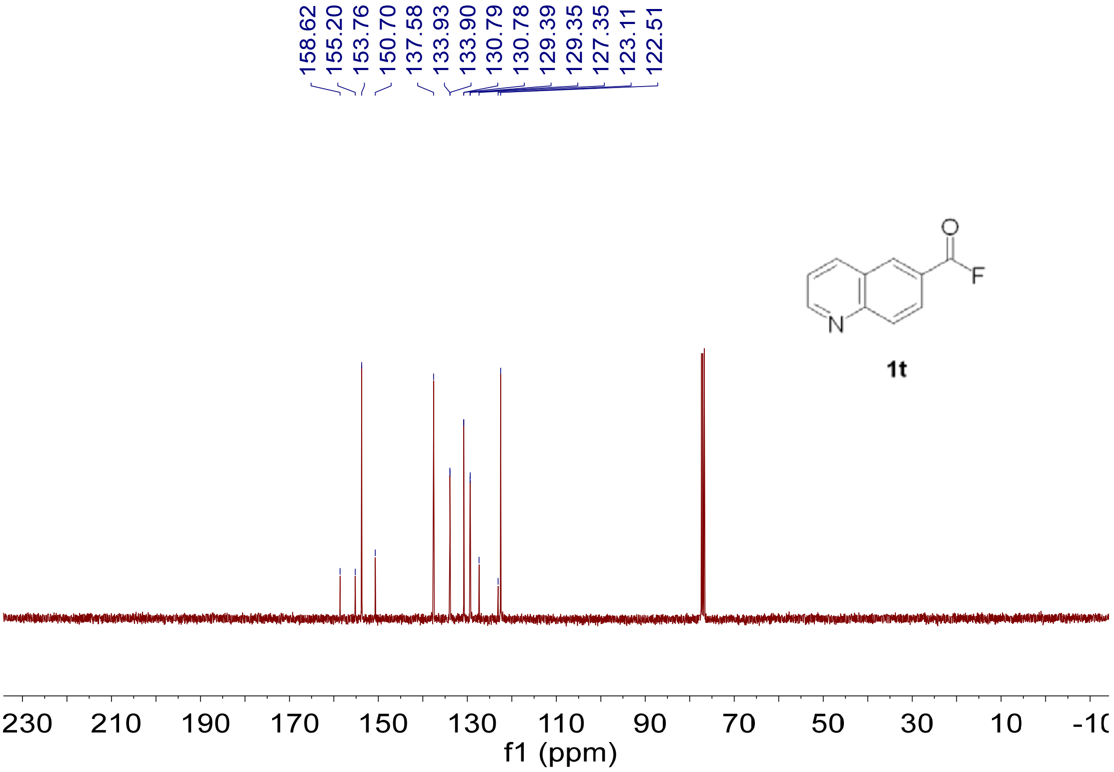

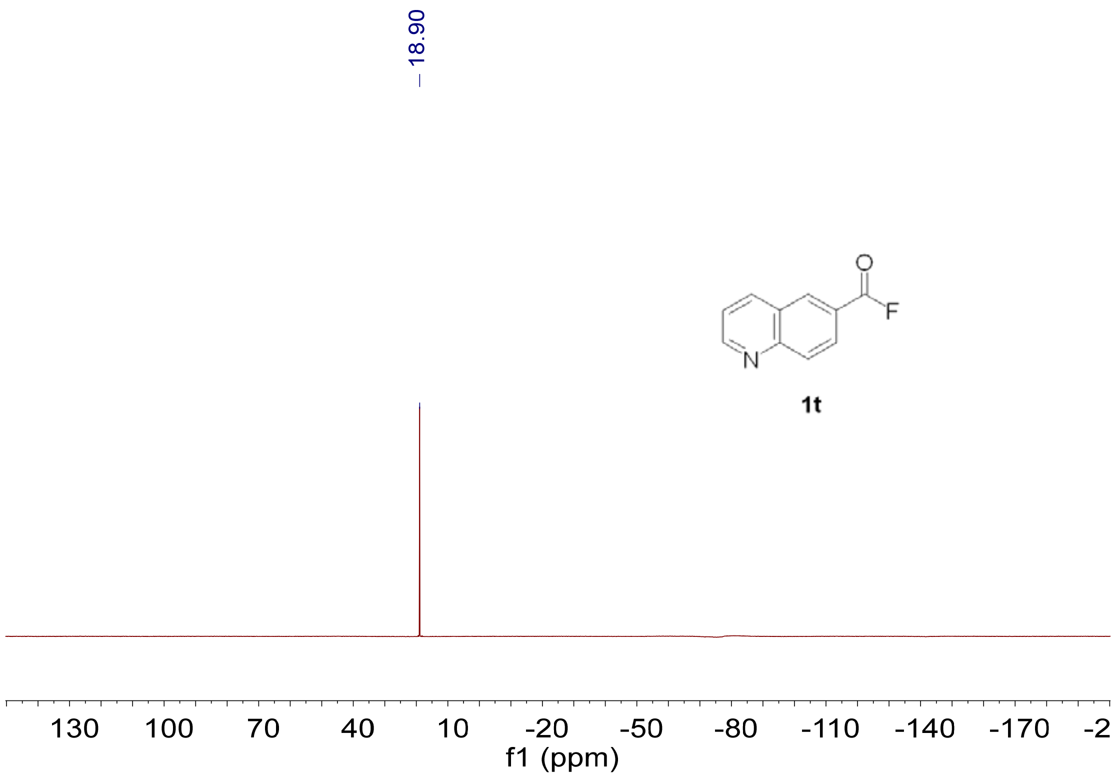

^{1}H NMR (400 MHz), ^{13}C{^{1}H} NMR (151 MHz) and ^{19}F{^{1}H} NMR (282 MHz) spectra of **1t** ($CDCl_3$, rt).

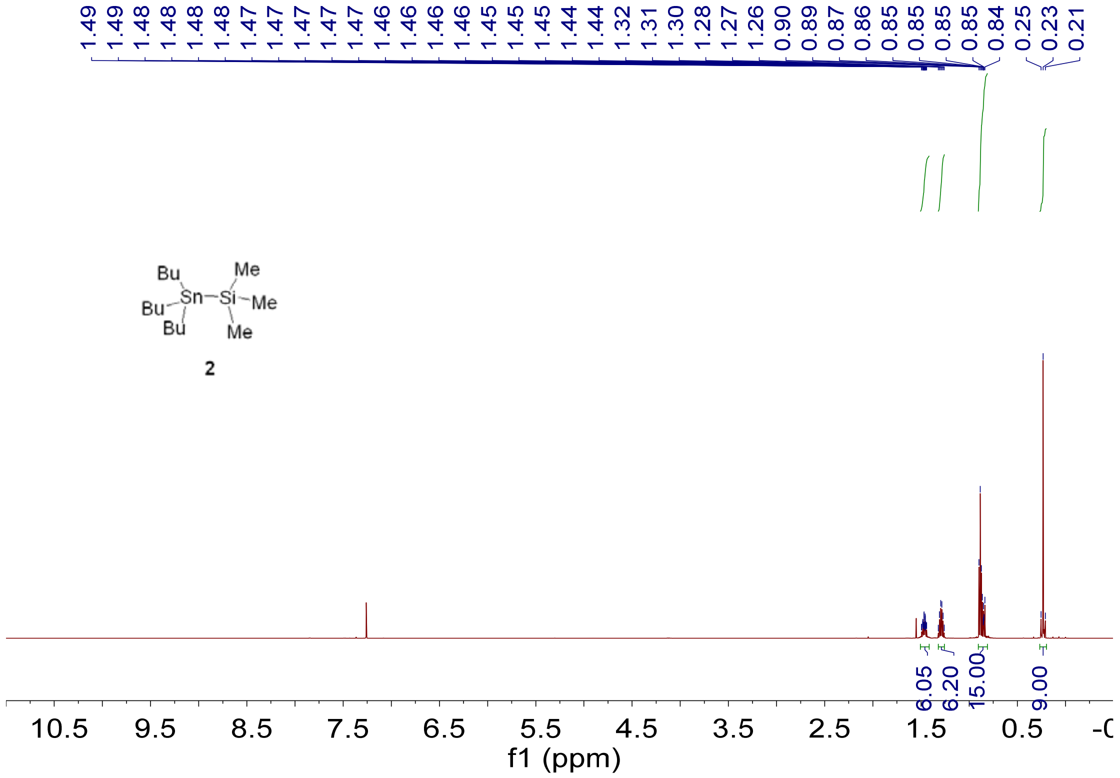

^{1}H NMR (600 MHz) spectrum of **2** ($CDCl_3$, rt).

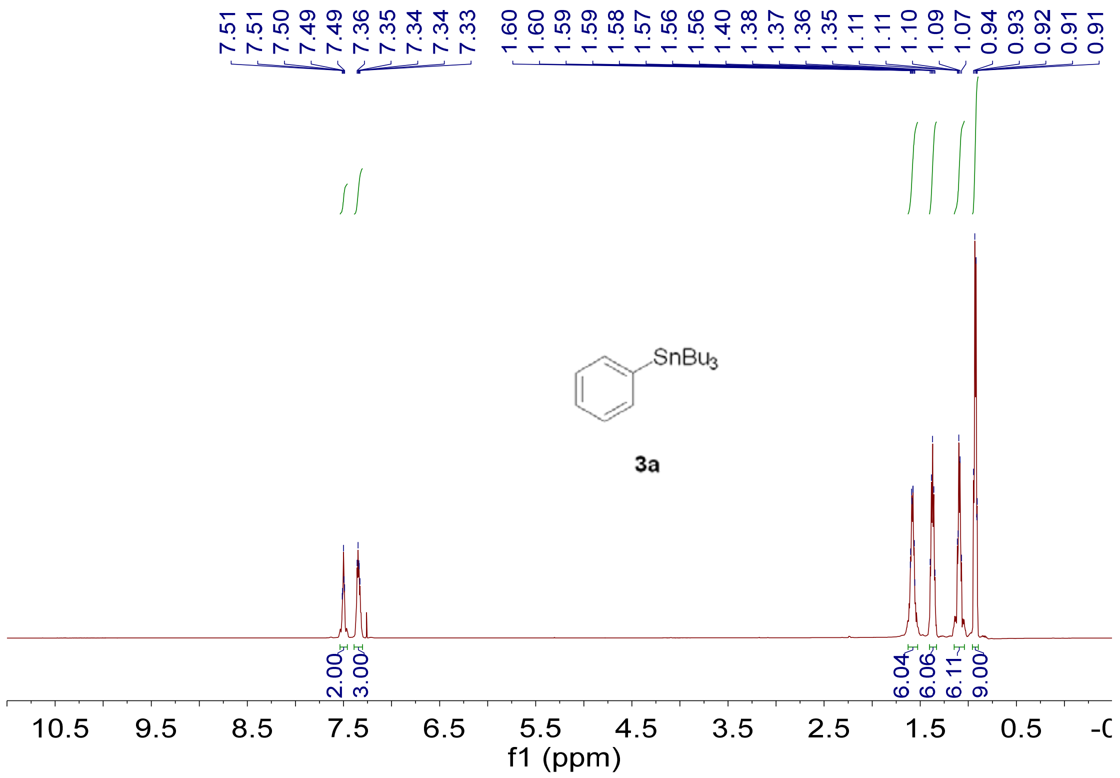

^{1}H NMR (600 MHz) spectrum of **3a** ($CDCl_3$, rt).

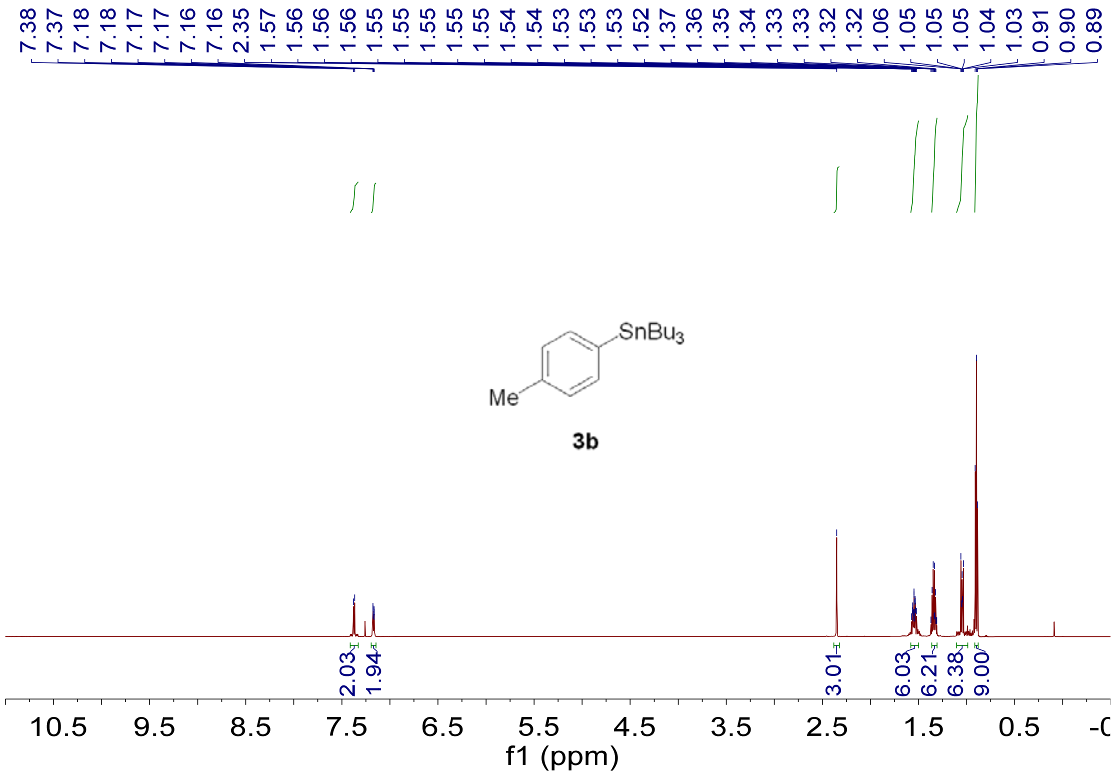

^{1}H NMR (600 MHz) spectrum of **3b** ($CDCl_3$, rt).

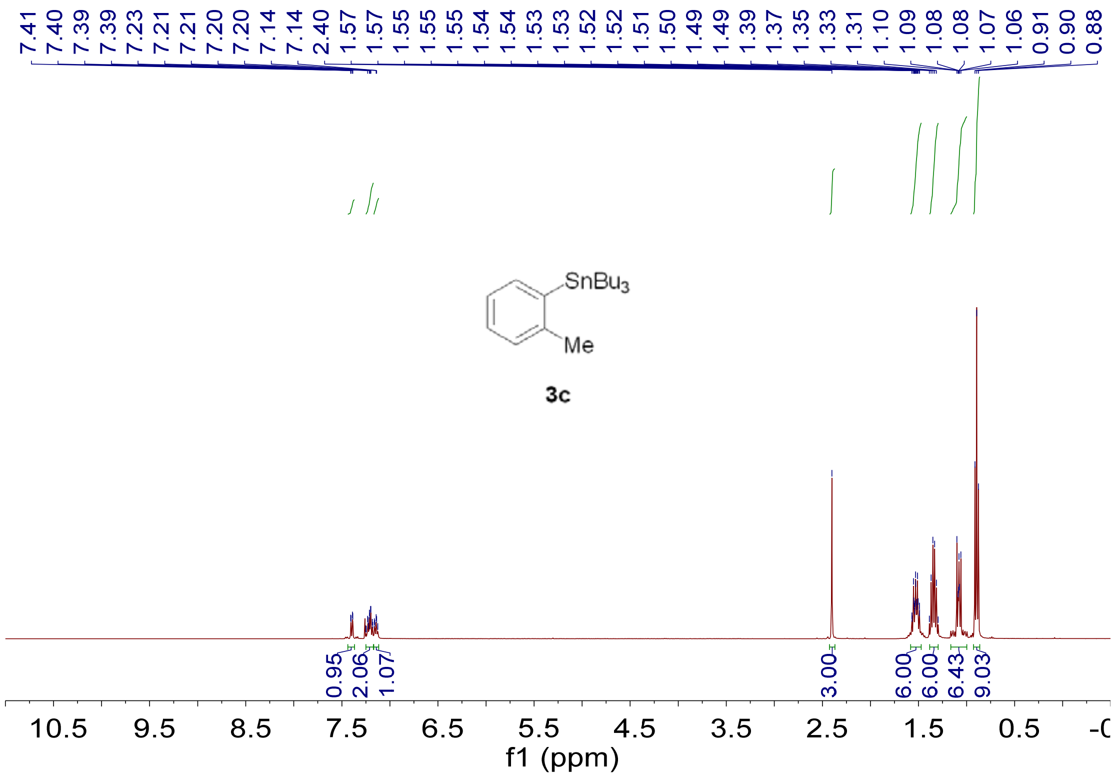

^{1}H NMR (400 MHz) spectrum of **3c** ($CDCl_3$, rt).

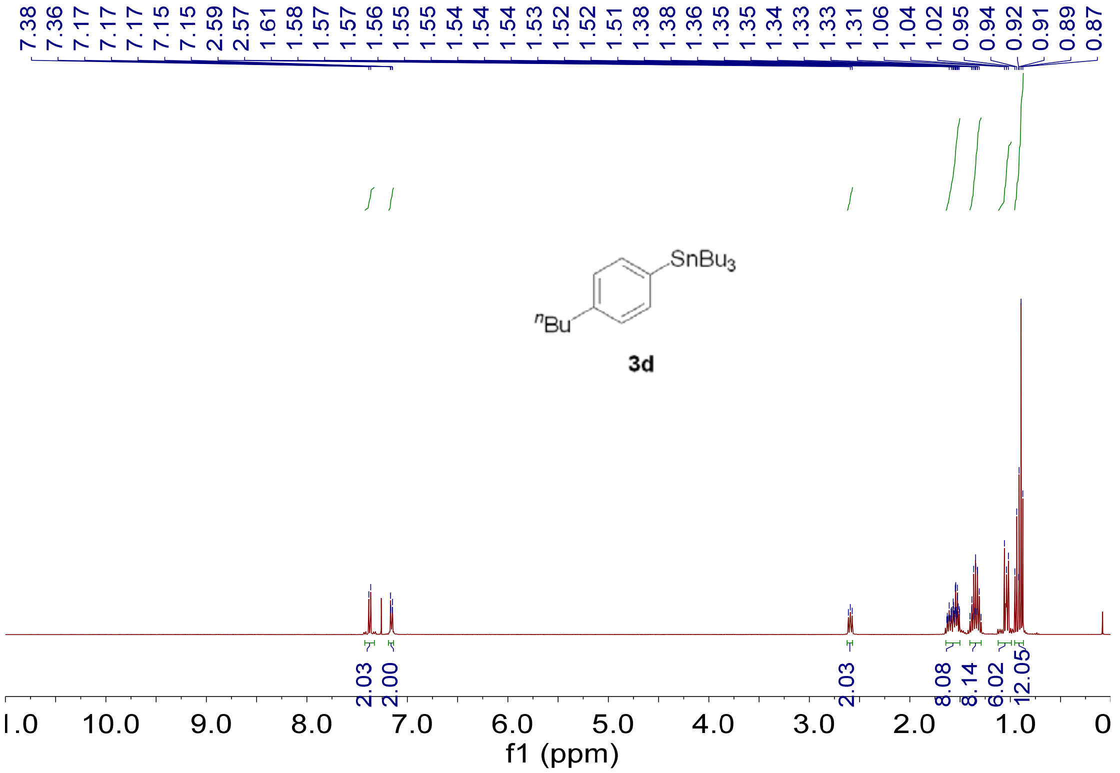

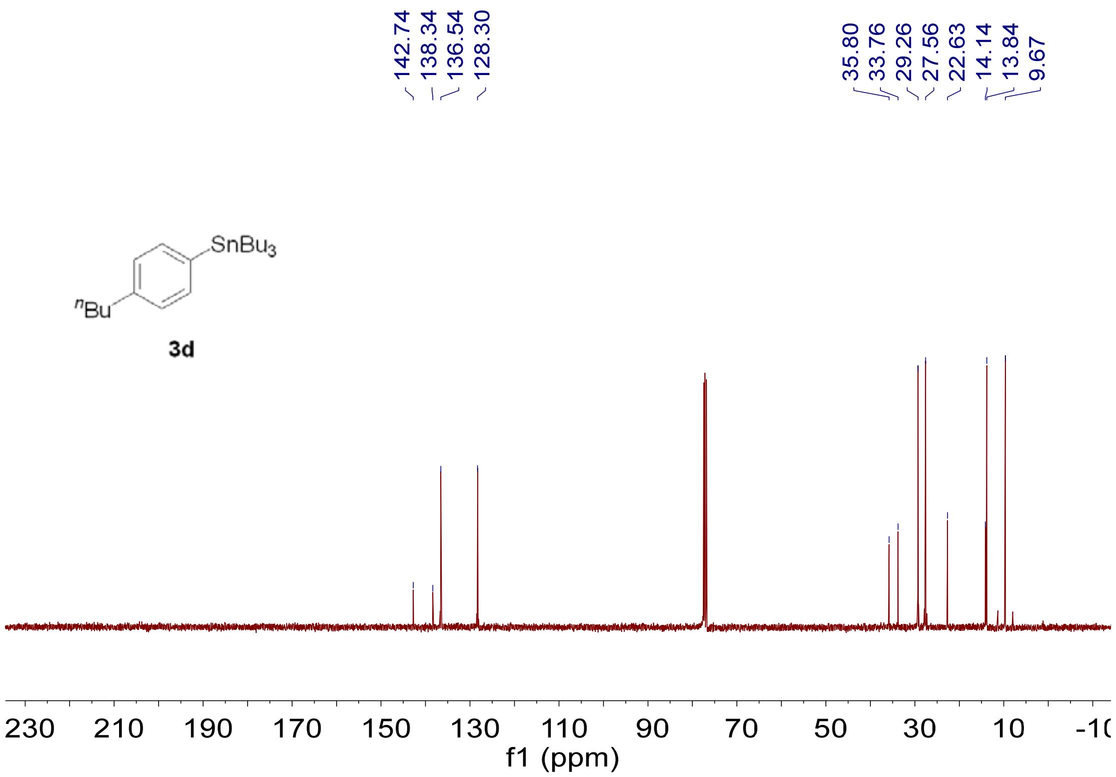

^{1}H NMR (400 MHz) and $^{13}C\{^{1}H\}$ NMR (101 MHz) spectra of **3d** ($CDCl_3$, rt).

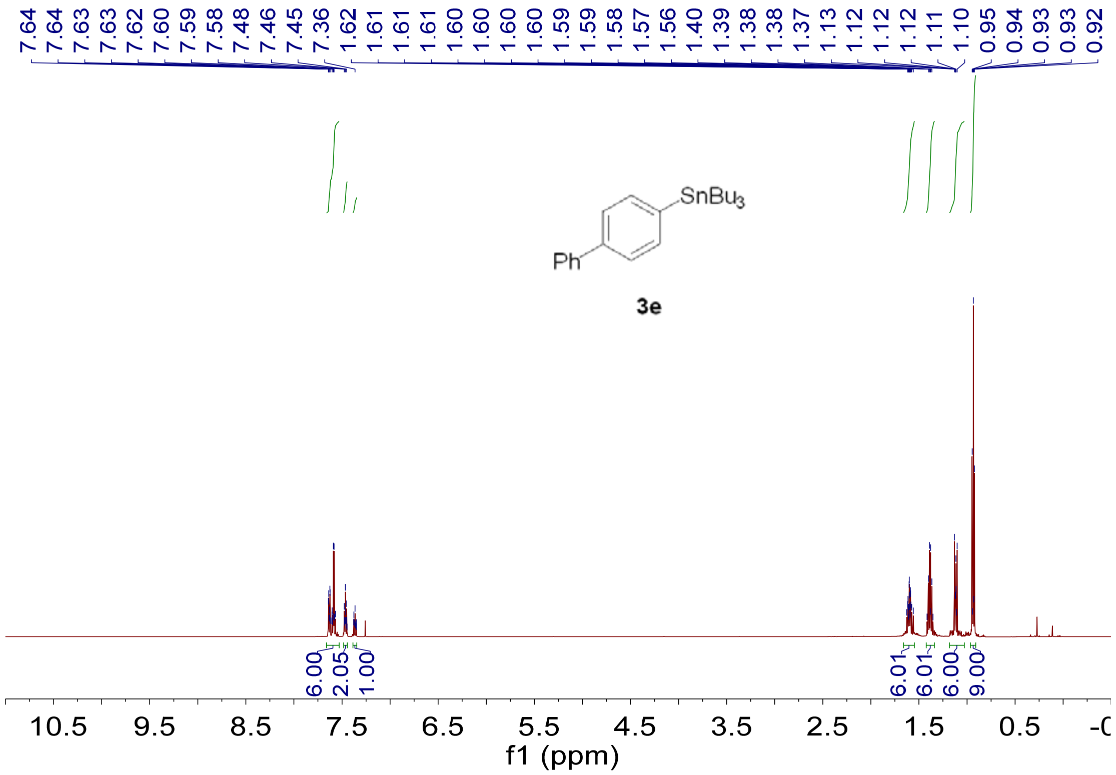

^{1}H NMR (600 MHz) spectrum of **3e** ($CDCl_3$, rt).

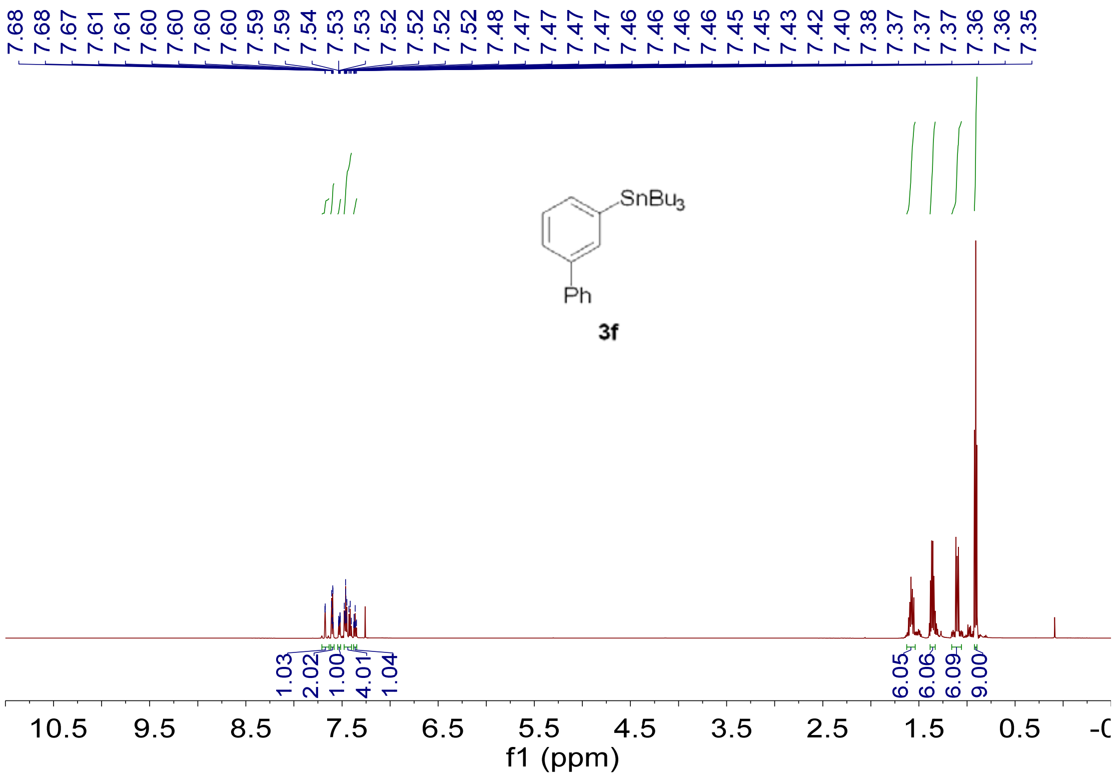

^{1}H NMR (600 MHz) spectrum of **3f** ($CDCl_3$, rt).

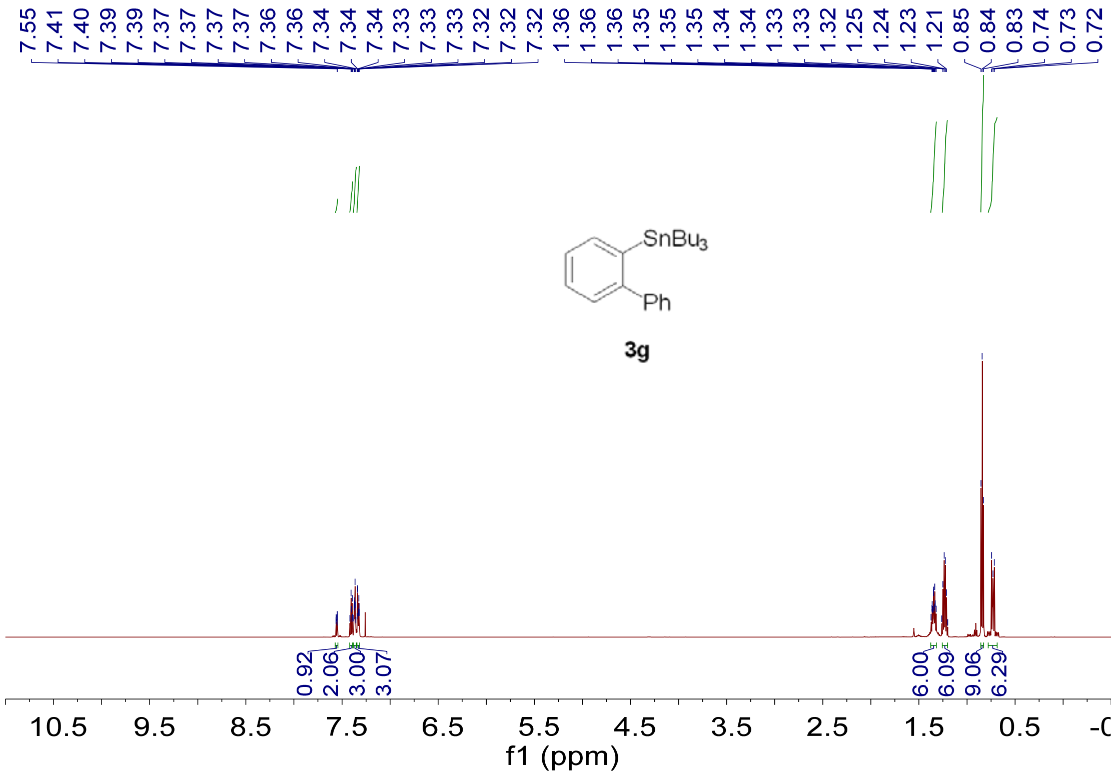

^{1}H NMR (600 MHz) spectrum of **3g** ($CDCl_3$, rt).

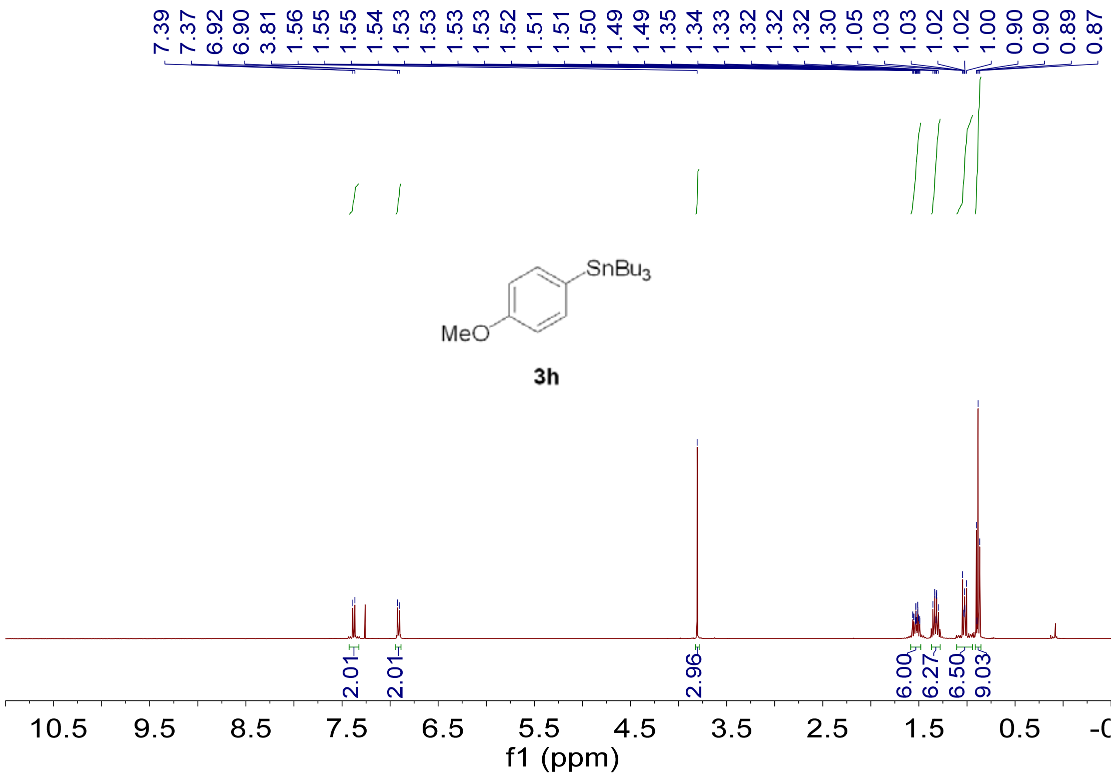

^{1}H NMR (400 MHz) spectrum of **3h** ($CDCl_3$, rt).

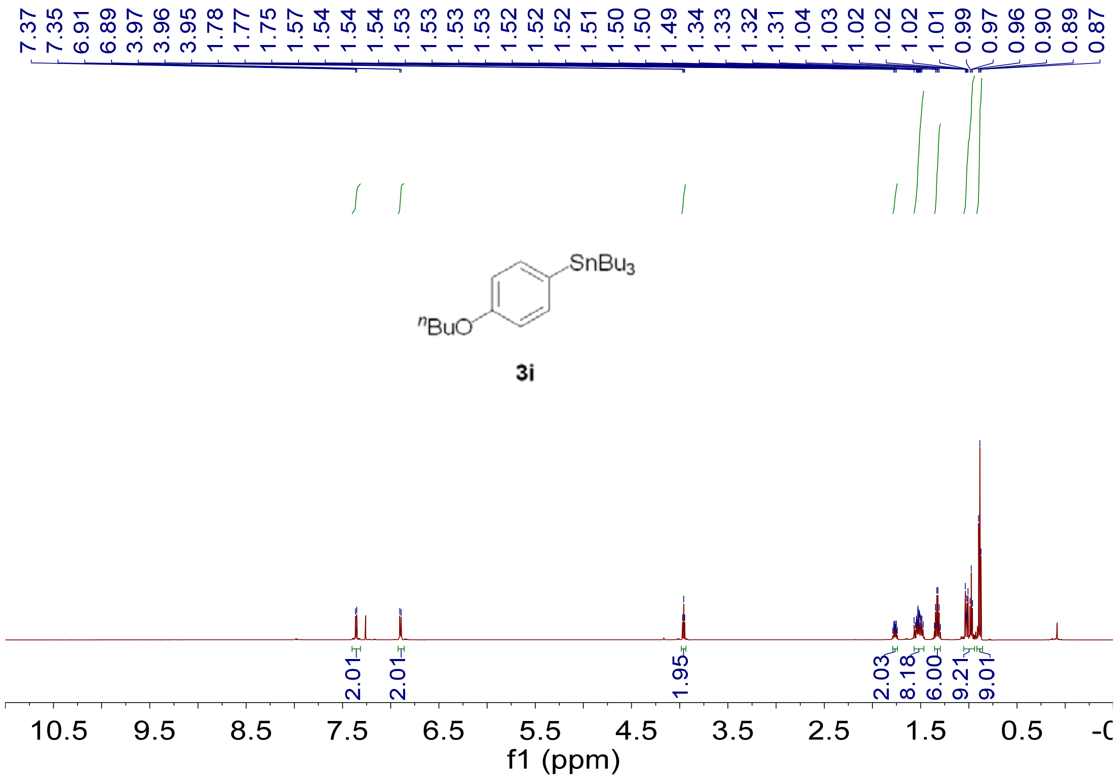

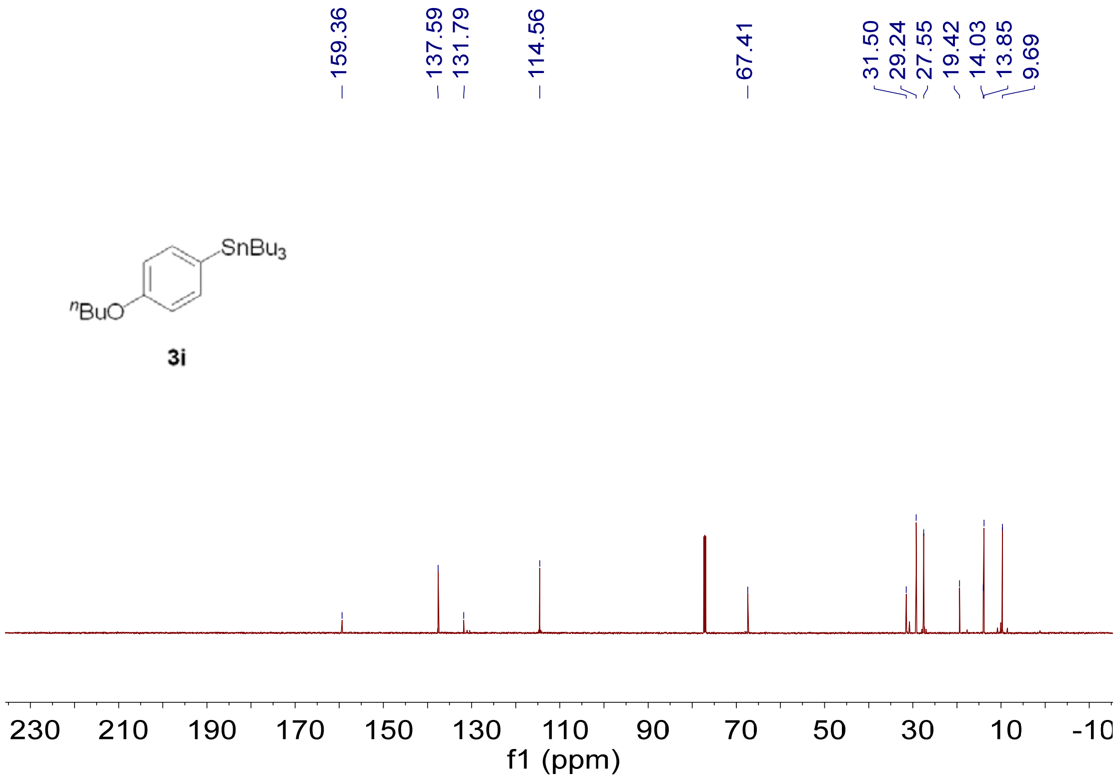

^{1}H NMR (600 MHz) and ^{13}C{^{1}H} NMR (151 MHz) spectra of **3i** ($CDCl_3$, rt).

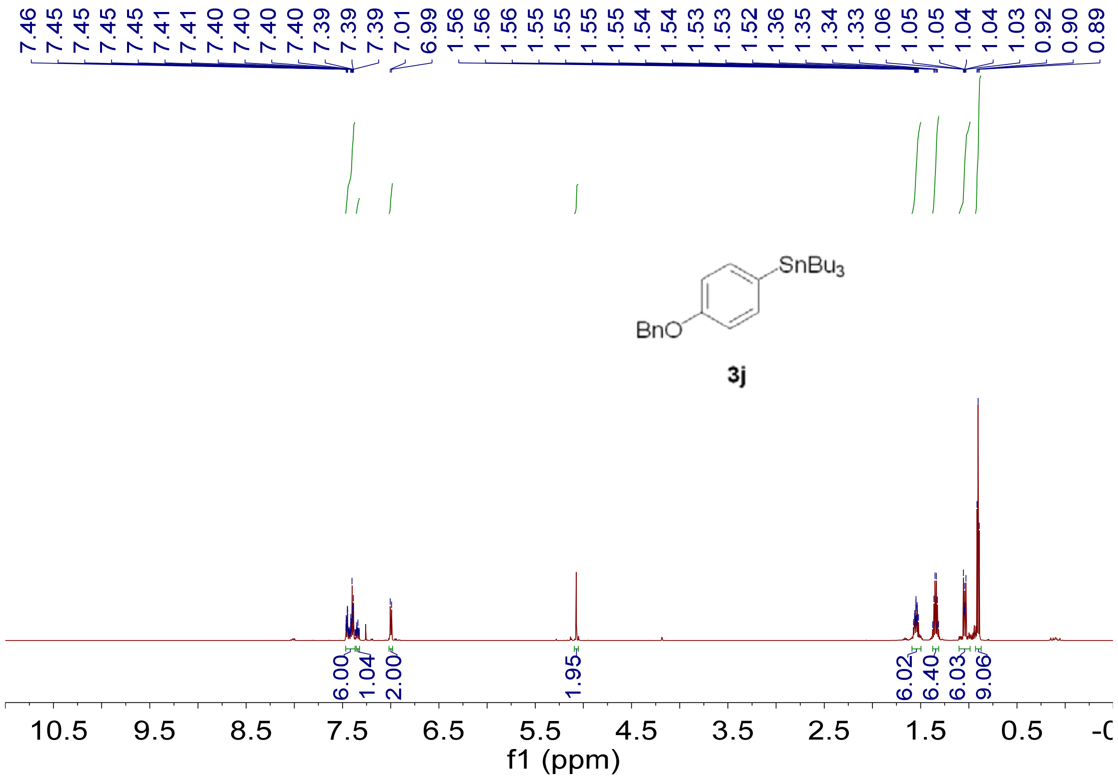

^{1}H NMR (600 MHz) spectrum of **3j** ($CDCl_3$, rt).

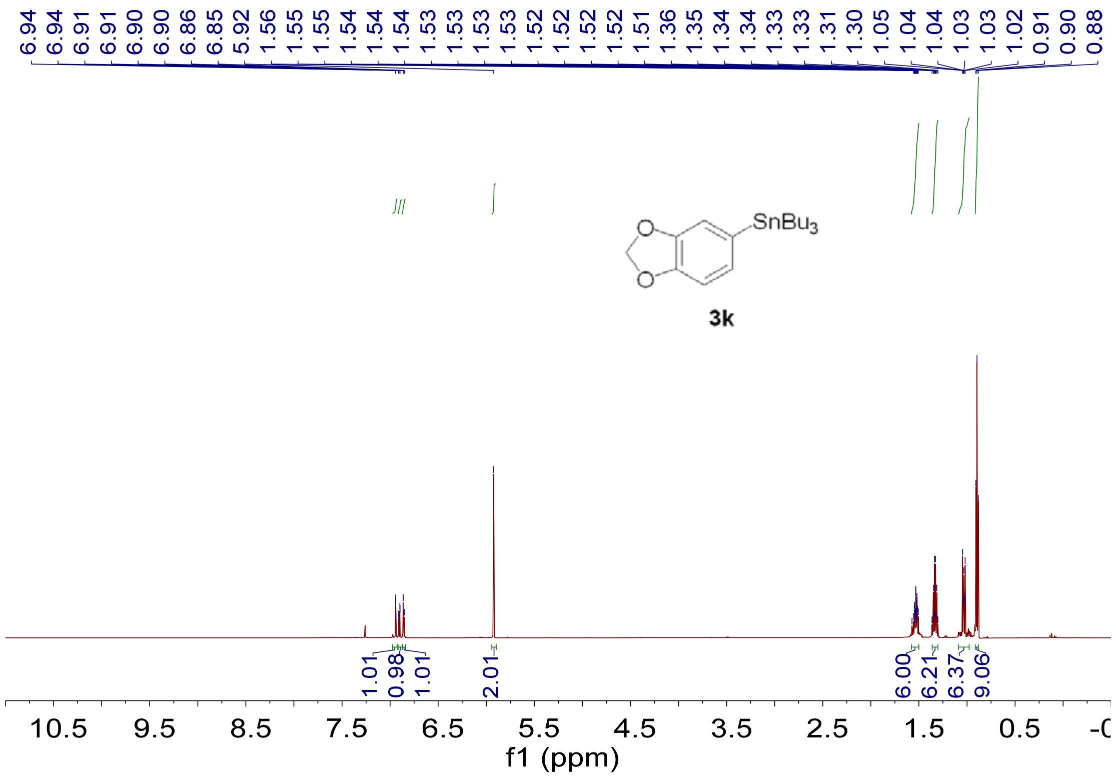

^{1}H NMR (600 MHz) spectrum of **3k** ($CDCl_3$, rt).

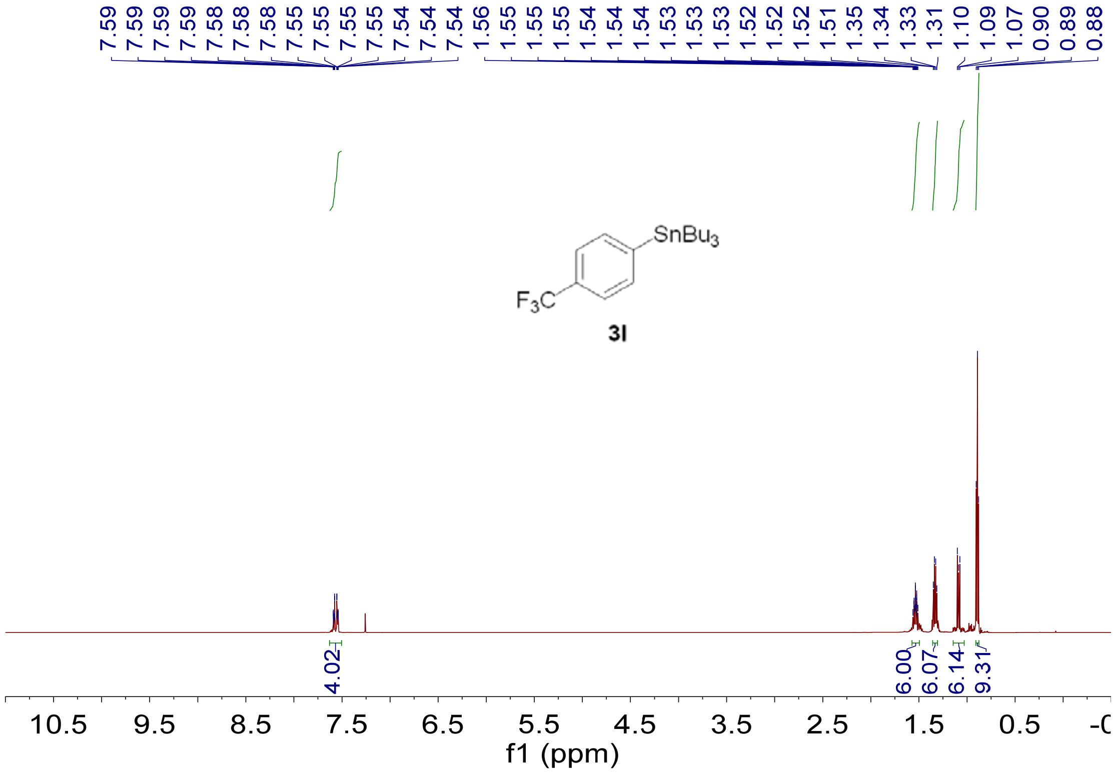

^{1}H NMR (600 MHz) spectrum of **3l** ($CDCl_3$, rt).

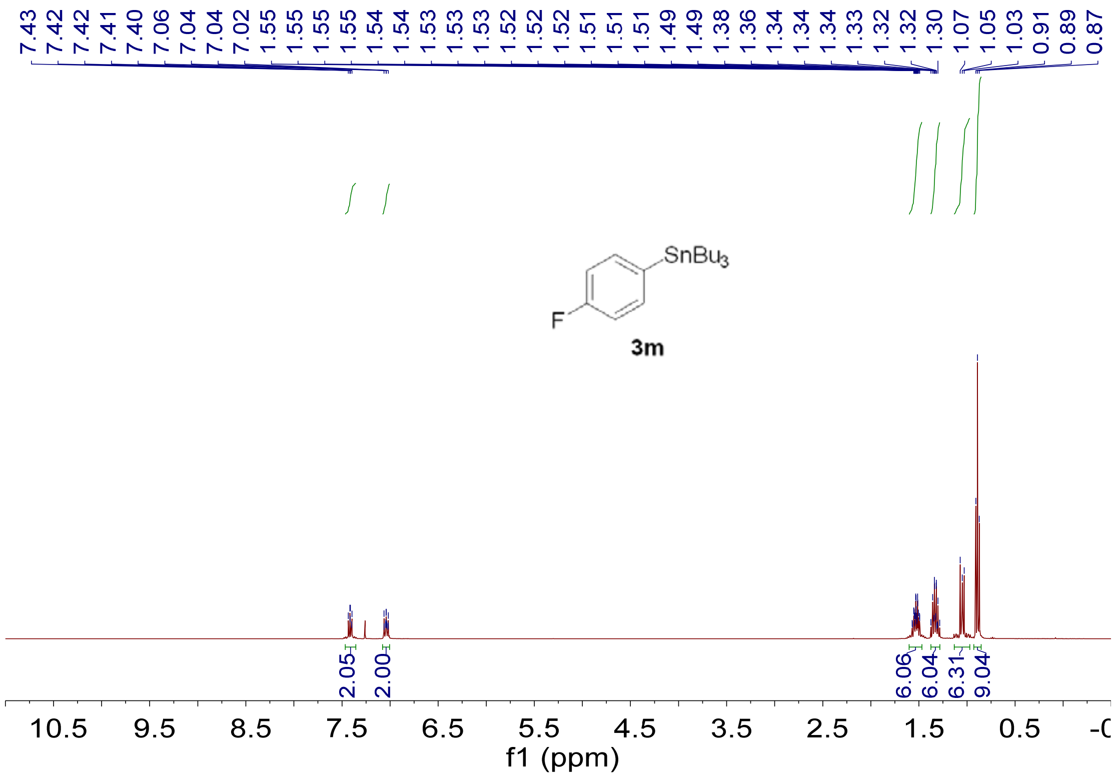

^{1}H NMR (400 MHz) spectrum of **3m** ($CDCl_3$, rt).

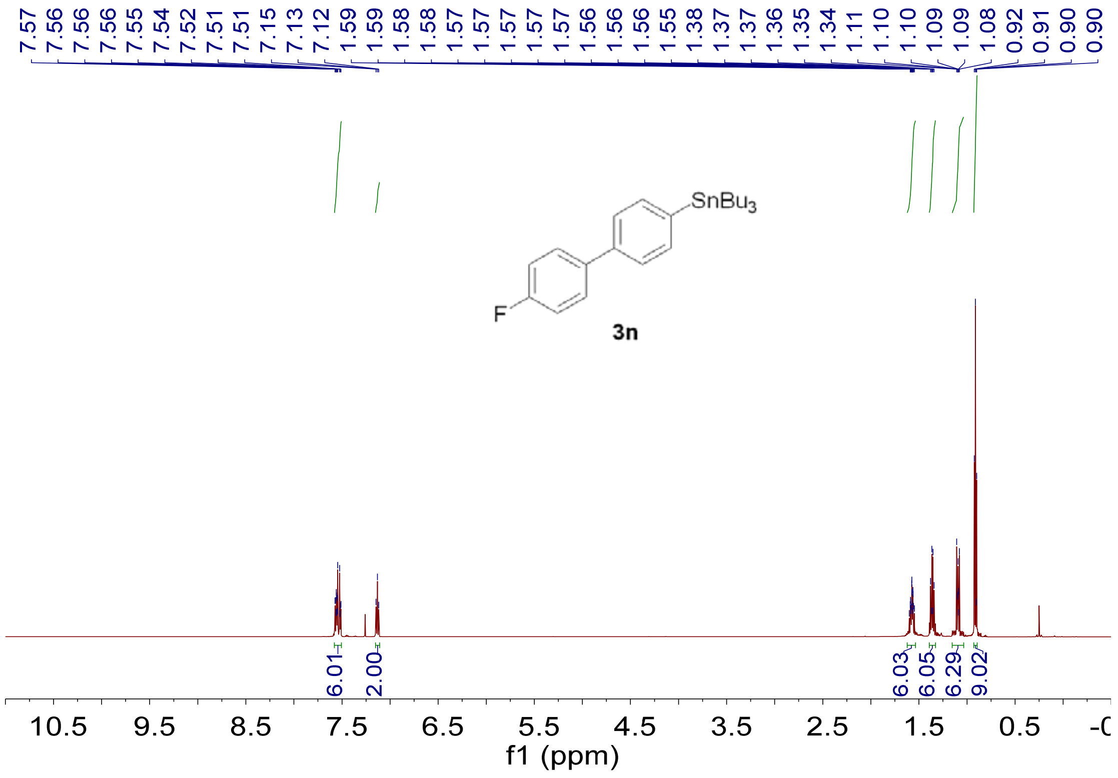

^{1}H NMR (600 MHz) spectrum of **3n** ($CDCl_3$, rt).

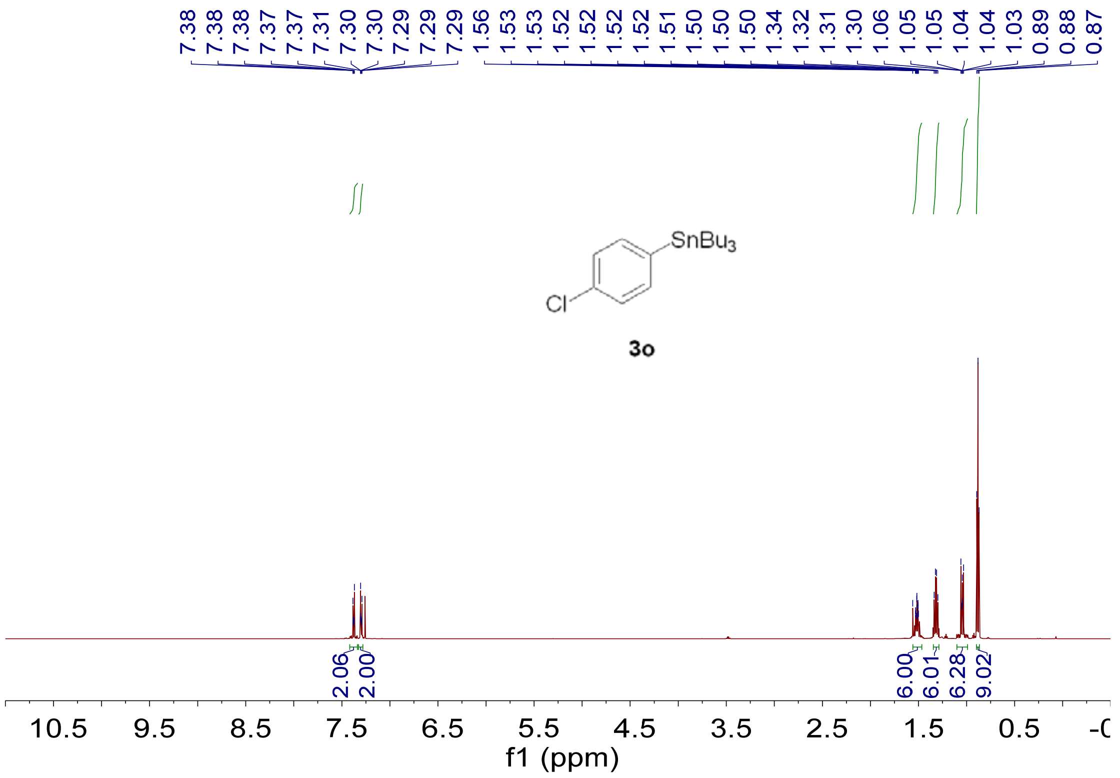

^{1}H NMR (600 MHz) spectrum of **3o** ($CDCl_3$, rt).

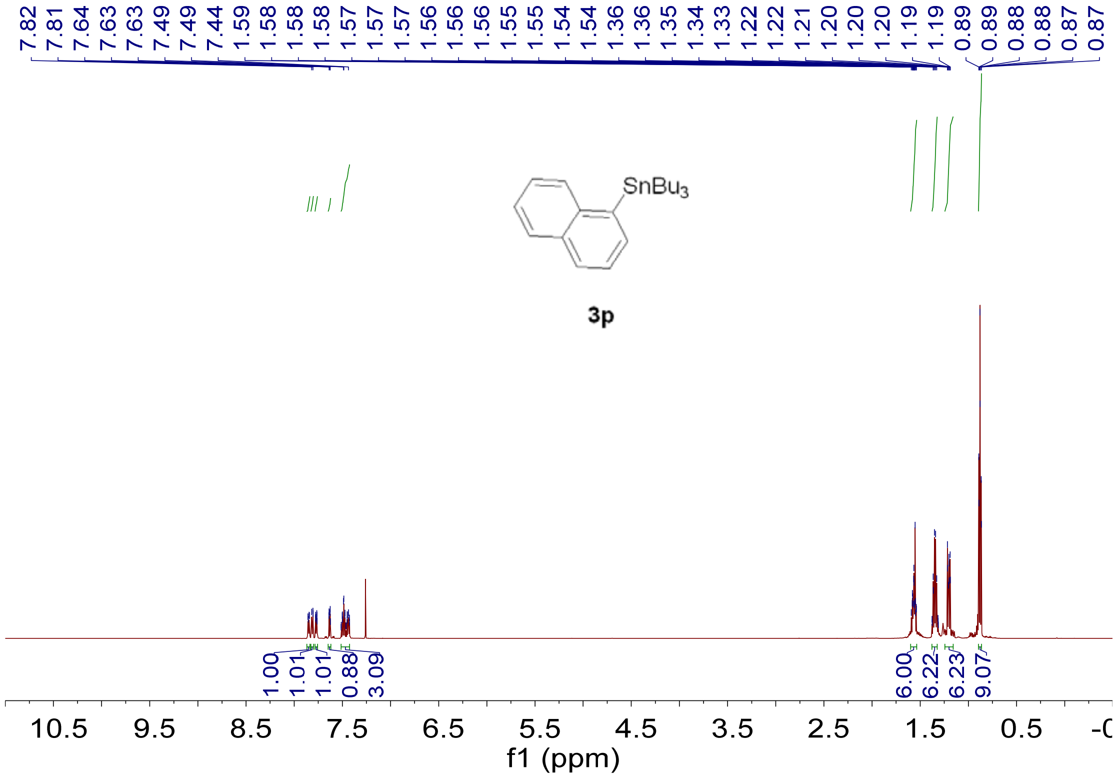

^{1}H NMR (600 MHz) spectrum of **3p** ($CDCl_3$, rt).

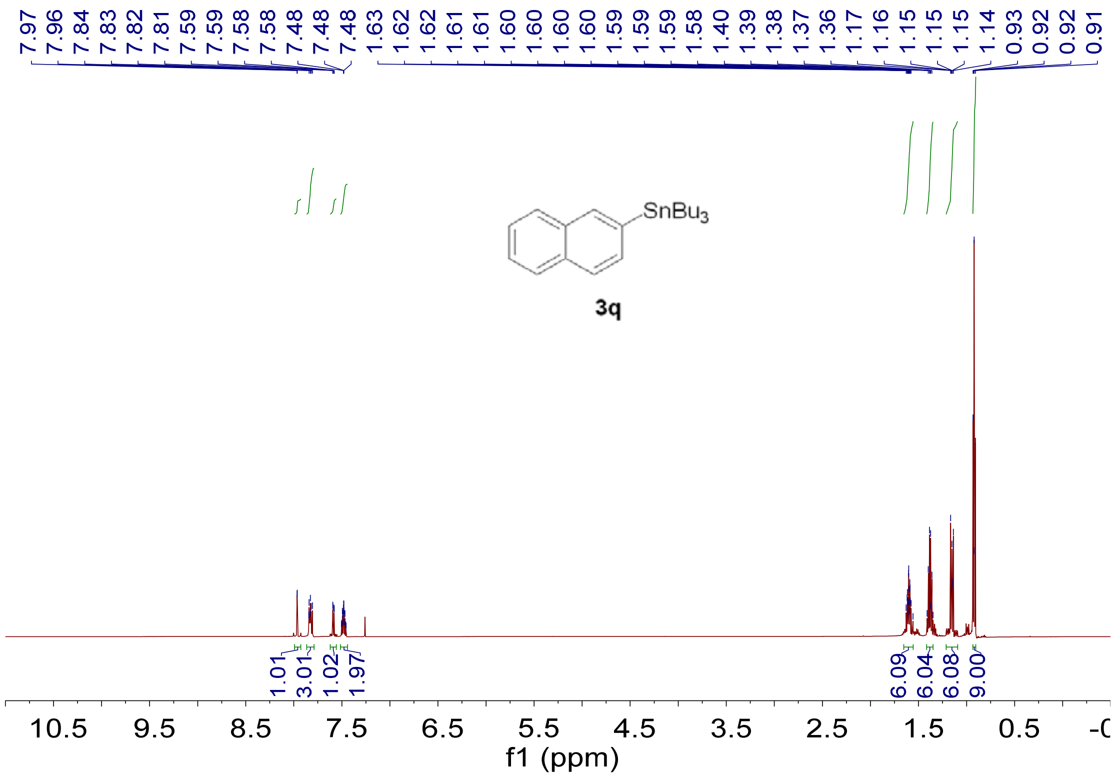

^{1}H NMR (600 MHz) spectrum of **3q** ($CDCl_3$, rt).

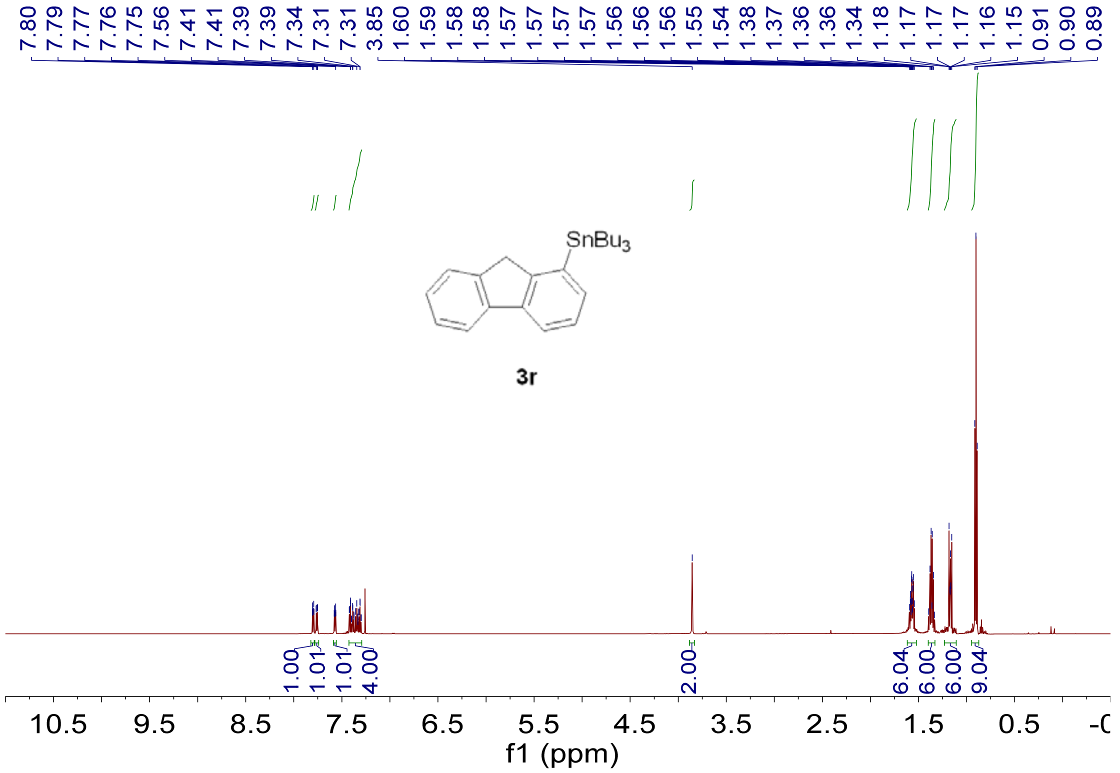

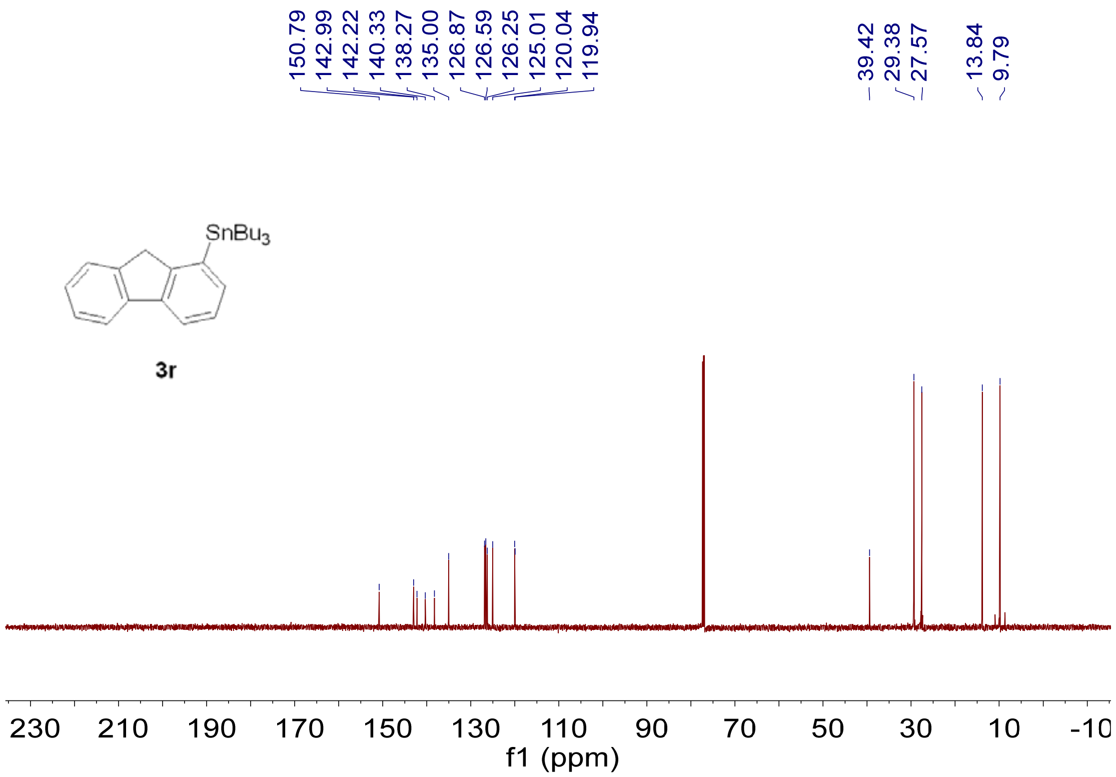

^{1}H NMR (600 MHz) and ^{13}C{^{1}H} NMR (151 MHz) spectra of **3r** ($CDCl_3$, rt).

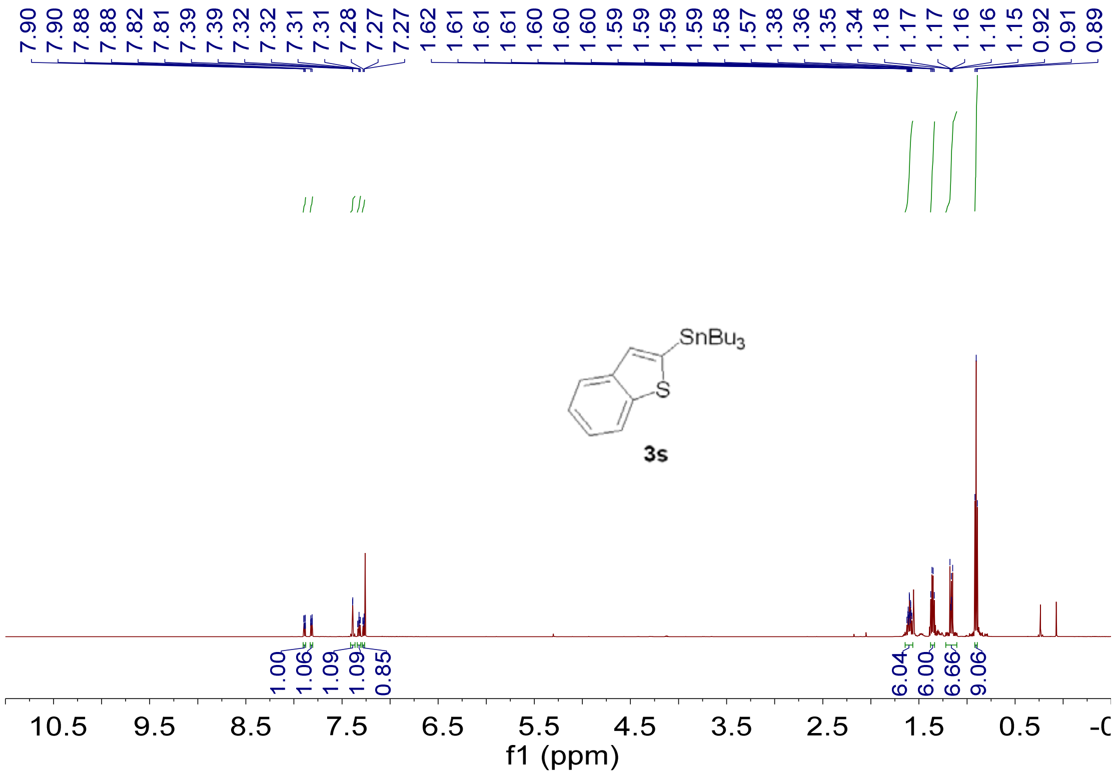

^{1}H NMR (600 MHz) spectrum of **3s** ($CDCl_3$, rt).

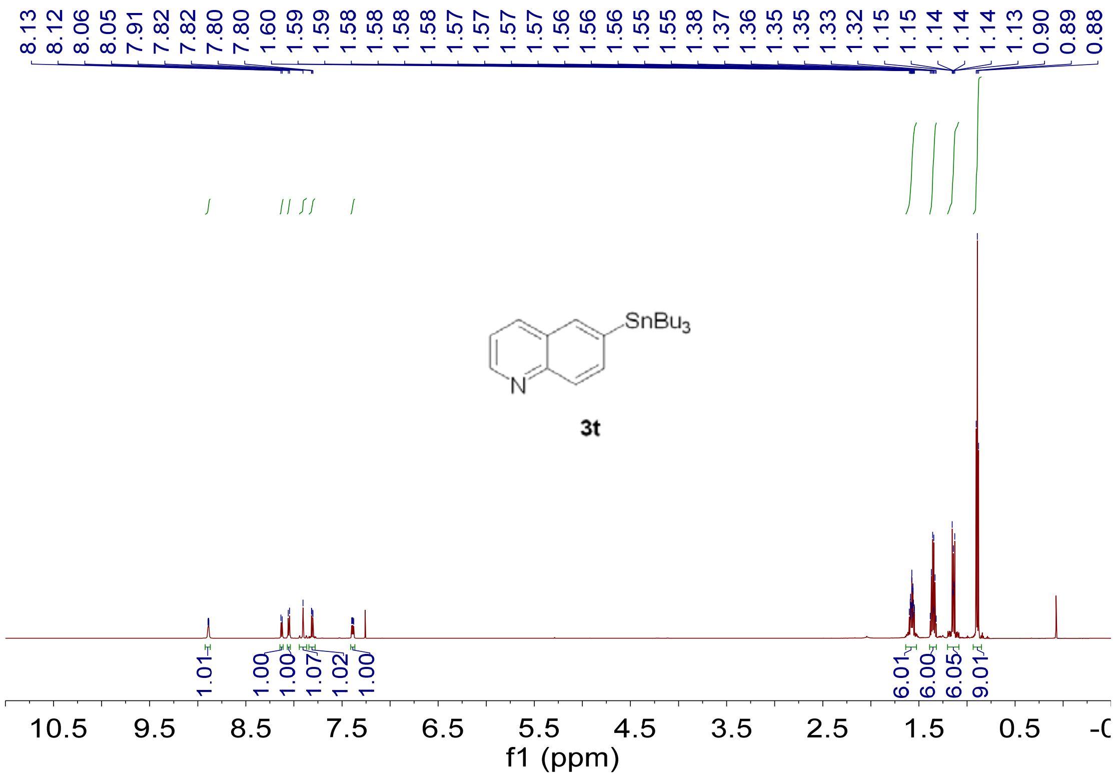

^{1}H NMR (600 MHz) spectrum of **3t** ($CDCl_3$, rt).

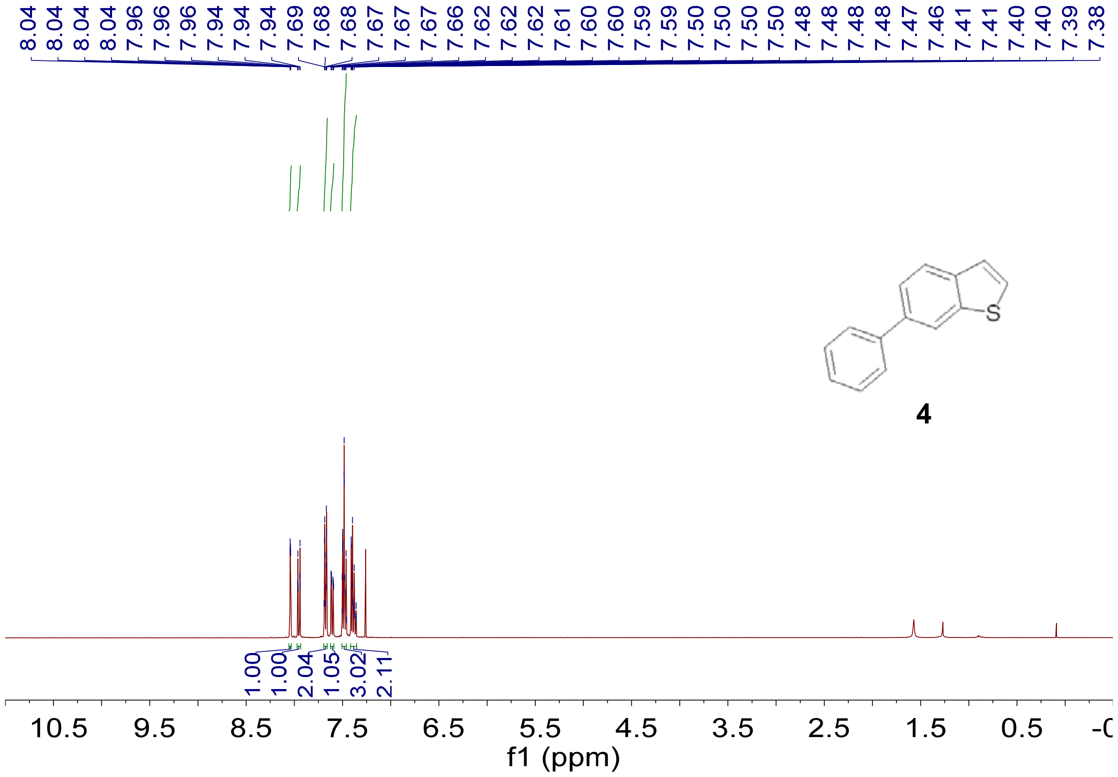

^{1}H NMR (600 MHz) spectrum of **4** ($CDCl_3$, rt).
